# Opposing effects of spatiotemporal variation in resources and temporal variation in climate on density dependent population growth in seabirds

**DOI:** 10.1111/1365-2656.13819

**Published:** 2022-10-31

**Authors:** Kate R. Searle, Adam Butler, James J. Waggitt, Peter G. H. Evans, Maria I Bogdanova, N. Thompson Hobbs, Francis Daunt, Sarah Wanless

**Affiliations:** ^1^ UK Centre for Ecology & Hydrology Edinburgh UK; ^2^ Bioinformatics and Statistics Scotland Edinburgh UK; ^3^ Bangor University Bangor UK; ^4^ Sea Watch Foundation Amlwch UK; ^5^ Natural Resource Ecology Laboratory, Department of Ecosystem Science and Sustainability & Graduate Degree Program in Ecology Colorado State University Fort Collins Colorado USA

**Keywords:** climate, density dependence, population dynamics, population growth rate, resource heterogeneity, spatiotemporal variation

## Abstract

1. Understanding how ecological processes combine to shape population dynamics is crucial in a rapidly changing world. Evidence has been emerging for how fundamental drivers of density dependence in mobile species are related to two differing types of environmental variation—temporal variation in climate, and spatiotemporal variation in food resources. However, to date, tests of these hypotheses have been largely restricted to mid‐trophic species in terrestrial environments and thus their general applicability remains unknown.
2. We tested if these same processes can be identified in marine upper trophic level species. We assembled a multi‐decadal data set on population abundance of 10 species of colonial seabirds comprising a large component of the UK breeding seabird biomass, and covering diverse phylogenies, life histories and foraging behaviours.
3. We tested for evidence of density dependence in population growth rates using discrete time state‐space population models fit to long time‐series of observations of abundance at seabird breeding colonies. We then assessed if the strength of density dependence in population growth rates was exacerbated by temporal variation in climate (sea temperature and swell height), and attenuated by spatiotemporal variation in prey resources (productivity and tidal fronts).
4. The majority of species showed patterns consistent with temporal variation in climate acting to strengthen density dependent feedbacks to population growth. However, fewer species showed evidence for a weakening of density dependence with increasing spatiotemporal variation in prey resources.
5. Our findings extend this emerging theory for how different sources of environmental variation may shape the dynamics and regulation of animal populations, demonstrating its role in upper trophic marine species. We show that environmental variation leaves a signal in long‐term population dynamics of seabirds with potentially important consequences for their demography and trophic interactions.

## Graphical abstract

How might environmental variation shape population regulation? We find that density dependent feedbacks to population growth are strengthened by climatic temporal variation and weakened by spatiotemporal variation in resources. This environmental variation leaves a signal in long‐term population dynamics of marine top predators, with important consequences for population ecology.

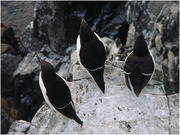

## 1 INTRODUCTION

Understanding fundamental mechanisms shaping species' distributions and dynamics has formed a key challenge in modern ecology, particularly for understanding how populations are structured and regulated. Population regulation can occur through processes such as density dependence, but such processes may themselves be affected by spatial and temporal changes to habitat and climate (Ehrlen & Morris, 2015; Owen‐Smith, 2014). Density dependence is a key regulator of populations, driving important feedbacks on age‐specific survival and fecundity, which are key determinants of population size and change over time (Dennis & Taper, 1994). Understanding this interplay requires a holistic approach to identify how properties of ecological systems, such as density dependence, resource variation and climate, together shape demographic processes (Coulson et al., 2001; Forchhammer et al., 1998; Giroux et al., 2014; Grenfell et al., 1998; Herrando‐Perez et al., 2012; Wang et al., 2006; Wang et al., 2013).

An emerging tenet from terrestrial macroecological studies, primarily on large herbivores, is that temporal variation in climate strengthens density‐dependent feedbacks to population growth (e.g., Chamaille‐Jammes et al., 2008; Coulson et al., 2001; Wang et al., 2006). In contrast, spatial variation in food resources weakens feedbacks (Wang et al., 2006, 2009). Animals experiencing strong climate variation may incur negative effects from both higher physiological stress increasing energetic demands, and through lowered overall carrying capacity inducing greater competition for resources, leading to stronger density dependence in populations when the relative decrease in carrying capacity is greater than any concurrent relative decrease in the intrinsic rate of population increase. In contrast, higher spatiotemporal variation in food resources may lead to improved animal nutrition and fitness through tracking of resource phenology, facilitating access to higher quality resources over prolonged time periods (i.e. phenological development of resources occurring at different times in different parts of the foraging landscape; Hebblewhite et al., 2008; Mysterud et al., 2001; Pettorelli et al., 2003; Searle et al., 2015). Such greater spatiotemporal variation in food resources may act to buffer animals against challenging conditions, weakening the strength of density dependence in populations.

Density‐dependent regulation in populations has been explored in marine systems including studies on fish, seals and some seabirds (Barbraud et al., 2018; Fromentin et al., 2001; Goyert et al., 2017; Horswill et al., 2017; Lu et al., 2020; Rotella et al., 2009; Zabel et al., 2006). However, it is the interplay between demographic and environmental processes that will ultimately influence population dynamics in variable environments. Although aspects of these differing sources of environmental variation on population processes have been explored within some marine populations (Barbraud & Weimerskirch, 2003; Descamps et al., 2015), and more rarely across a suite of marine species (Barbraud et al., 2018; Goyert et al., 2017), we currently lack a test across multiple populations and species, spanning several decades, to understand the generality of these processes. Such a test would significantly enhance support for these drivers of population processes, if they are found to occur in marine systems as has been demonstrated in terrestrial studies.

Temporal variation in climate and spatial variation in food resources are both sources of heterogeneity that may be expected to influence population regulation in mobile predators such as seabirds. Strong variation in climate over time, including extreme events, reduces breeding success and survival in a range of species, including some seabirds (Diamond et al., 2020; Frederiksen et al., 2008; Newell et al., 2015). Fluctuating climate may push animals beyond their physiological limits affecting energy balance and fitness, as well as decreasing carrying capacity in some years, potentially through bottom up effects propagating up marine food webs and reducing prey availability (Frederiksen et al., 2006), or through more direct reductions in habitat such as loss of breeding sites during storms (Newell et al., 2015) or periods of high snow or ice cover (Chastel et al., 1993). Therefore, it is possible that the same amplification of density dependent effects on population growth rates documented in terrestrial mid‐trophic species may also be found in marine upper‐trophic seabirds. Weakening of density dependent effects on seabird population growth rates with increasing spatial variation in food resources could arise from variation in phenological development of prey resources, affecting availability and quality, particularly during the breeding season (Rindorf et al., 2000). Therefore, if foraging environments for seabirds exhibit greater spatiotemporal variation in prey resources, birds may be able to achieve greater access to the most profitable prey types at key stages in the seasonal cycle. Thus, for a given overall resource amount, we may expect seabirds with access to more spatiotemporal variation in prey resources to achieve higher nutrition through prolonged access to high quality prey over key stages in the season, ameliorating density dependent effects driven by competition at higher population densities.

Strong links between prey abundance and seabird demography have been established, identifying a threshold in prey abundance below which seabirds experience consistently reduced and more variable productivity (Cury et al., 2011), but evidence for a link between spatiotemporal variation in prey resources and seabird demographic rates is generally lacking because of the paucity of long‐term data sets on seabirds and their prey (see Crawford, 2007 for review). In addition, studies of density dependence in marine birds have predominantly focused on relationships for single colonies or species (Ashbrook et al., 2010; Frederiksen & Bregnballe, 2000) (but see Barbraud et al., 2018; Goyert et al., 2017; Horswill et al., 2017). Our work tested these relationships across the UK seabird community, occupying the temperate northeast Atlantic, primarily the North Sea, Celtic Seas and English Channel. This is a region that has undergone major changes in both marine climate and prey resources over recent decades (Frederiksen et al., 2013; Mitchell et al., 2020). We studied ten seabird species (Table 1), comprising around 40% of the total number of breeding seabird species in the UK, and approximately two‐thirds of the breeding pairs (estimated from Seabird 2000 Census data, Mitchell et al., 2004). The majority of seabirds in this region forage during the breeding season on small, shoaling, lipid‐rich fish, principally lesser sandeel *Ammodytes marinus*, sprat *Sprattus sprattus*, larval or juvenile herring *Clupea harengus* or juvenile gadids (Mitchell et al., 2004), and there is strong population structure amongst forage fish species, with large‐scale spatiotemporal patterns in growth, maturation and fecundity (MacDonald et al., 2015). This region represents one of the best‐studied marine ecosystems in the world, for which there is accumulating evidence for trophic and climatic interactions (Carroll et al., 2015; Frederiksen et al., 2006; Lauria et al., 2013). However, knowledge of the prevalence of density dependent demographic processes and their relationship with environmental variation remains limited (Horswill et al., 2017). We first tested for the prevalence and strength of direct density dependence on population growth rate in our suite of seabird species. We then sought to understand how environmental variation may have shaped direct density dependence within these species. We developed discrete time state‐space models for seabird population dynamics to examine (1) how temporal variation in climate and spatiotemporal variation in prey resources shape the presence and strength of density dependence in UK seabird species over the last four decades, and (2) if the same characteristics of environmental variation affecting population processes in mid‐trophic level species in terrestrial systems are also operating in this marine predator community.

**TABLE 1:** Number of breeding colonies with greater than 20 years with counts for each species used in the population models. Year range gives overall time period across all colonies per species, estimated UK breeding population size (counts of pairs for all species except common guillemot, black guillemot and razorbill, which are counts of individuals) from JNCC seabird 2000 census data (summarised in Mitchell et al., 2004).

Species	Number of colonies meeting data requirements	Year range	UK breeding population size
Common guillemot *Uria aalge*	20	1985–2014	1,416,334
Northern fulmar *Fulmarus glacialis*	29	1985–2015	501,609
Black‐legged kittiwake *Rissa tridactyla*	36	1985–2015	378,847
Razorbill *Alca torda*	21	1985–2015	187,052
Lesser black‐backed gull *Larus fuscus*	12	1985–2015	111,960
Arctic tern *Sterna paradisaea*	9	1969–2014	53,380
Black guillemot *Cepphus grylle*	12	1982–2015	38,714
European shag *Gulosus aristotelis*	25	1986–2015	26,565
Great black‐backed gull *Larus marinus*	15	1985–2015	16,755
Common tern *Sterna hirundo*	20	1969–2014	11,838

## 2 MATERIALS AND METHODS

### 2.1 Data

We collated count data from breeding colonies for the period 1986 to 2016 from the UK Seabird Monitoring Programme (SMP, available at http://jncc.defra.gov.uk/smp/) for ten seabird species. These ten species were selected because they are abundant in the study system (Table 1), include representatives from the main seabird families (Procellaridae, Phalacrocoradidae, Alcidae, Laridae), and span different breeding and foraging strategies. We selected breeding colonies with at least 20 years of count data (Supplementary Material S1). Species‐specific count methodology is given in Walsh et al. (1995). Counts were of breeding pairs for all species except Common Guillemot *Uria aalge*, Razorbill *Alca torda* and Black Guillemot *Cepphus grylle*, where the count unit was individuals (see Supplementary Material S2 for method of converting these into breeding pairs). The SMP employs a range of count methods depending on species and location, and some colonies or ‘sub‐sites’ listed in the SMP are in fact only partial colonies, for instance sections along a cliff (Walsh et al., 1995). Therefore, to standardise selection of breeding colonies to use in model fitting we dropped sub‐sites listed in the SMP with very low numbers, defined as those at which the maximum count was less than the 10% sample quantile calculated over maximum counts from all colonies for each species. We also dropped colonies at which the first observed count was less than the 5% sample quantile across the first observed counts from all colonies for each species (Supplementary Material S1). Similarly, because some breeding colonies had sporadic counts prior to the start of the SMP (1986), where there was a gap greater than 5 years between the first count and the subsequent count, we dropped the first year of observation from the time series. Applying the data requirements to the collated breeding colony counts resulted in between nine and 36 breeding colonies selected for population modelling for each species (Table 1). The use of these data, or any part of our analysis, did not require ethical approval.

### 2.2 Statistical modelling

For each time series of counts *y*_1_, …, *y*_*n*_ we fitted three alternative process models to assess evidence for density dependence: a model with no density dependence (null, M0), a model with direct density dependence (of lag 1 year, M1) and a model with direct and delayed density dependence (of lag 1 year and lag 2 years, M2). The two density dependence models (M1 and M2) incorporated density dependence using the discrete time stochastic version of the Ricker logistic growth equation (Dennis & Taper, 1994; Ricker, 1954). We note that other authors have used a discrete time stochastic version of the Gompertz growth equation when detecting density dependence in time series for animal populations (e.g., Wang et al., 2013). However, the Gompertz formulation assumes that growth rate depends only logarithmically on population density, which may introduce a loss of predictive ability (Dennis & Taper, 1994). Similarly, the logistic version we use here offers a more flexible choice for modelling the dynamic behaviour of populations (Dennis & Taper, 1994).

Counts of animals have inherent measurement errors that may bias estimation of the strength of density dependence (Freckleton et al., 2006), so we embedded the population model within the Bayesian state‐space framework. Within the state‐space population model for each time series, the observed count *y*_*t*_ was assumed to be related to the unobserved true population size *N*_*t*_ through an ‘observation model’; the dynamics of the population size *N*_*t*_ over time was then described by one of the three ‘process models’ described above. The parameters of the observation and process models were estimated simultaneously within a Bayesian framework via Markov chain Monte Carlo (MCMC) (Clark & Bjornstad, 2004).

For most species, the observed counts, *y*_*t*_, were assumed to have a Poisson distribution conditional upon the true (unobserved) abundance *N*_*t*_, so that $y_{t}\sim \text{Poisson}\left(N_{t}\right)$. The range of methods employed to estimate population size (*y*_*t*_) in the SMP (Walsh et al., 1995) means that there is no obvious model for the error structure. In the absence of detailed information on the error structure associated with individual records in the dataset, and because the sampling methods mean that both under and overestimation of counts are possible, we adopt a Poisson model for observation error. For Common Guillemot and Razorbill, the raw counts related to individuals, not breeding pairs, so we assumed instead that $y_{t}\sim \text{Poisson}\left(K_{t}N_{t}\right)$, where the true, unobserved, annual adjustment from individuals to breeding pairs, *K*_*t*_, is assumed to come from a normal distribution: $K_{t}\sim \text{normal}\left(m_{\mathrm{kt}},s_{\mathrm{kt}}^{2}\right)$, with mean equal to the mean smoothed value of the conversion $\left(m_{\mathrm{kt}}\right)$ and standard error equal to the standard error $\left(s_{\mathrm{kt}}\right)$ associated with this smoothed value (Supplementary Material S2). The smoothed values of the conversion from individuals to breeding pairs in each year were estimated using generalised additive models fitted to empirical estimates of the annual adjustment (Harris, Heubeck, et al., 2015; Harris, Newell, et al., 2015; Supplementary Material S2). For black guillemot this adjustment was not available, so the unadjusted counts were modelled as individuals.

Two process models for the dynamics of the true (unobserved) number of breeding pairs $N_{t}$ in year t were fitted; the two models represent variations in the assumed form of population regulation. The first model (M0) assumes that growth is independent of density, and that inter‐annual stochastic variation follows a log‐normal distribution, such that $N_{t}=N_{t-1}\exp\left(\beta_{0}+\varepsilon_{t}\right)$, where process error $\varepsilon_{t}\sim \text{normal}\left(0,\sigma_{\varepsilon }^{2}\right)$. The standard deviation of the residual process error, $\sigma_{\varepsilon }$, was allowed to vary across each population within each species. The second model (M1) incorporates direct density dependence via the stochastic discrete time logistic growth model (Dennis & Taper, 1994): $N_{t}=N_{t-1}\exp\left(\beta_{0}+\beta_{1}N_{t-1}+\varepsilon_{t}\right)$. Note that M0 is a special case of M1 (in which $\beta_{1}=0$), and that parameters $\beta_{0},\beta_{1}$ were colony specific.

The parameter $\beta_{0}$ represents the intrinsic rate of population increase at each colony (analogous to ‘*r*’ in the classic Ricker equation) such that $e^{\beta_{0}}$ is the discrete time growth rate customarily denoted as lambda (*λ*). The parameter $\beta_{1}$ represents the strength of direct density dependence at each colony (the additive change in the log of per capita population growth rate per change in *N*_*t*_, analogous to −*r*/*K* in the Ricker equation). If parameter $\beta_{1}$<0 the per‐unit‐abundance growth rate decreases as *N* becomes larger. An increasing per‐unit‐abundance growth rate, or Allee effect, arises when parameter $\beta_{1}$>0 (Dennis & Taper, 1994).

We set an informative prior on parameter $\beta_{0}$ the intrinsic rate of population growth at each colony. Previous work has demonstrated that fitting density‐feedback models without prior information gives biologically unrealistic estimates for population growth rate in most cases, even in models as simple as the Ricker logistic (Delean et al., 2013). State space Ricker models formulated to be informative in this way using allometric relationships in a test on empirical and simulated data across 36 species were ranked higher (using Deviance Information Criterion) than (70% of species tested) or equivalent to (30% of species tested) models fitted using vague priors (Delean et al., 2013). Moreover, these models consistently outperformed models with vague priors when abundance time series had high standard deviations (Delean et al., 2013), as is most often the case in seabird time series. Therefore, incorporating prior knowledge of species' life history has been shown to provide more ecologically realistic estimates for population demography and to improve overall model fit, and is recommended practice for fitting density‐feedback models (Delean et al., 2013). However, recognising that informative priors for population growth rates may affect posterior estimates (Delean et al., 2013), we chose very conservative values for upper and lower bounds for this prior for all species. We therefore assumed that the per capita population growth rate for each species could never be greater than half the maximum clutch size *c* (Supplementary Material Table S3), which implies that all eggs are fledged and there is no mortality, and assumed that annual survival could not be lower than u (which was assumed equal to 0.2 for all species).

We then assumed that the parameter was uniformly distributed between these limits, such that: $\beta_{0}\sim \text{Uniform}\left(\log\left(u\right),\log\left(1+\frac{c}{2}\right)\right)$. In model M1, we assigned informative priors to the first one or two unobserved time points by setting a uniform prior with range equal to the range of the first four observed counts in each time series. All other priors were assumed to be diffuse; parameters $\beta_{0}$ and $\beta_{1}$ were assumed to have uniform priors bounded between −10 and 10. The process standard deviation, ($\sigma_{\varepsilon }$), was assigned a uniform prior bounded between 0 and 2. Note that the prior is assigned to the process standard deviation, so this bounding to lie between 0 and 2 implies the assumption that abundance may vary from year to year by approximately 50‐fold (because the exponential of two times the upper limit of the SD is approximately 50, although we note the level of expected variation will also depend upon population size and parameters $\beta_{0}$ and $\beta_{1}$). Missing data (SMP annual population counts) were treated as stochastic random variables within the models, and were estimated during model fitting as parameters of the state‐space model.

All models were fitted using JAGS (Plummer, 2003), utilising the ‘jagsUI’ package (Kellner & Kellner, 2017) and r (R Core Team, 2017). Marginal posterior distributions of model parameters and missing data were approximated using the Gibbs Markov chain Monte Carlo (MCMC) algorithm. Three chains of between 50,000 and 100,000 iterations (depending on species) were retained, after discarding between 20,000 and 30,000 iterations as burn‐in (species in which models were run for more iterations had burn‐ins at the upper end of this range). Chains were initialised with values diffuse from the mean of the priors. Convergence was assessed by monitoring the trace of the posteriors of variances and estimated parameters $\beta_{0,s}$, $\beta_{1,s}$ and *N*_*t*_, and by using the Gelman‐Rubin convergence statistic ($\hat{R}$) for each parameter as modified by Brooks and Gelman (1998). We used posterior predictive checks (Gelman, 2004) for each model and time‐series combination to determine if the fitted model could plausibly have given rise to the data. Bayesian *p* values were calculated by comparing the posterior predictive distribution for simulated data arising from the fitted model with the distribution of the observed count data (Hobbs & Hooten, 2015). This comparison was done by calculating the proportion of times that metrics derived from the observed values were greater than metrics derived from the simulated values, calculated across all MCMC samples. This proportion forms the Bayesian *p* value for each model, and should ideally lie close to 0.5. We then assessed the relative support in the data for each model using deviance information criterion (DIC), summed across all time series. The full mathematical expression for the posterior and fully factored joint distribution for each model is in Supplementary Material (S5).

To enable an approximate comparison of the strength of density dependence across different species, we calculated the strength of density dependence for a 10% increase in the mean population size per time series (Figure S4). This is because interpretation of parameter *β*_1_ in our model depends upon characteristics of the individual time‐series, in particular overall population abundance. We used the estimated multiplicative change in the discrete time population growth rate per change in population size and applied this to a 10% increase in the mean observed population size over each modelled time series. The strength of density dependence is therefore presented as the multiplicative proportional change in population growth rate, based upon the expected change in growth rate arising from a 10% increase in mean population size, *Nmean*_*t*_, derived as (exp[0.1 × *Nmean*_*t*_ × *β*_1_]), where *β*_1_ is the posterior mean from the fitted population model. Note that because this quantity relates to the multiplicative change in the growth rate, values close to 1 indicate weaker effects of density dependence, and values closer to zero represent stronger density dependent effects.

### 2.3 Relating density dependence to environmental variables

We ran a post hoc analysis to correlate the estimated strength of direct density dependence (lag year 1) with derived metrics for temporal variation in climate and spatiotemporal variation in food resources. We chose to use a two‐stage approach to be consistent with previous analyses (Wang et al., 2006, 2009), and minimised potential challenges relating to correct propagation of uncertainty from one analysis to the next by utilising the Bayesian framework. While this two‐stage approach may introduce some additional uncertainty, we were satisfied that normality of posterior distributions from the first stage was sufficient to warrant implementing the two‐stage method. For temporal variation in climate, we derived metrics for the standard deviation of sea surface temperature (mean potential temperature between 0 and 100 m; ‘SST’) and sea surface height (‘SSH’) over the period 1985–2016, spatially averaged around each colony up to a distance defined by the foraging range for each species (Table S6). Sea surface temperature has previously been linked to changes in seabird food resources (Frederiksen et al., 2013), therefore strong temporal variation in this climatic variable could be expected to affect density dependent processes via its mediating effect on prey availability and carrying capacity. Sea surface height is affected locally by wind speed and surface wind stress (Mishra, 2020; Sterlini et al., 2016), and local wind has been demonstrated as a dominant driver of sea surface height variation in the North Sea and NE Atlantic (Sterlini et al., 2016). We therefore included a metric capturing temporal variation in SSH as a proxy for temporal wind‐related climatic variation, known to affect the ability of seabirds to perform flight and foraging activities efficiently (Elliott et al., 2014; Pistorius et al., 2015; Richardson et al., 2018), thereby potentially contributing to a fluctuating carrying capacity for seabird populations, predicted to result in stronger density dependent effects. SST was sourced from FOAM AMM7 model outputs from the Marine Environment Monitoring Service (http://marine.copernicus.eu/) and processed at 10 km and monthly resolution. Daily SSH (m) was sourced from the National Oceanographic Centre Proudman Oceanographic Laboratory Coastal Ocean Modelling System (POLCOMS; https://www.bodc.ac.uk/data/documents/nodb/316641/) for the Atlantic margin of the northwest European shelf from 1985 to 2004 using a 1/9° latitude by 1/6° longitude grid with 40 s‐coordinate levels in the vertical (Holt et al., 2012; Wakelin et al., 2009). SSH data were processed at a 10 km daily resolution. Each metric was created by calculating the standard deviation in monthly values across only those months during which the species tend to associate most closely with their breeding colonies (April–July). These pixel level standard deviations were then averaged across all pixels within the foraging range of each species at each breeding colony to calculate the final metrics for temporal variation in climate, with one value per timeseries (Supplementary Material S6). Foraging ranges for each species were taken from recently published studies estimated from GPS tracking data (Thaxter et al., 2012; Wakefield et al., 2017; Table S6).

Relevant data for spatial and temporal variation in densities of key seabird prey species are unavailable for most seabirds, and proxies for prey such as oceanographic and climatic properties are used instead to define prey distribution and availability (Tremblay et al., 2009). Studies utilising GPS tracking data of breeding seabirds have been used to identify linkages between persistent patterns of seabird at‐sea distributions and their behaviour during the breeding season in relation to oceanographic variables such as bathymetry, productivity (chlorophyll), tidal fronts and eddy potential (for reviews see Tremblay et al., 2009; Waggitt et al., 2018; Wakefield et al., 2009), suggesting that these oceanographic‐climatic variables may be used as proxies for prey availability. For spatiotemporal variation in food resources, we used proxies previously positively associated with foraging habitat for seabirds—mean chlorophyll across 0 and 100 m (‘chlorophyll’) and an indicator of tidal fronts (‘tidal fronts’; Waggitt et al., 2018; Wakefield et al., 2009). Calculations of tidal front intensity and chlorophyll used FOAM AMM7 model outputs from the Marine Environment Monitoring Service, and were processed at 10 km and monthly resolution. To derive a metric capturing relevant spatiotemporal variation in resources for seabirds, we calculated the temporal standard deviation in each variable over time for the period 1985–2016, and then determined the spatial standard deviation across all pixels within the foraging range of each colony by species (Supplementary Material S6). These standard deviations were, as with the temporal climate variables, calculated over all breeding season months (Supplementary Material S6). We assumed that all covariates were estimated without error, and hence did not model their distributions.

To assess relationships between the strength of density dependence and environmental variation, we correlated these environmental metrics with the estimated strength of direct density dependence across all colonies within each species. We assumed the estimated direct density dependence at each colony *s* ($D.{\mathrm{est}}_{s}$ as the estimated strength of direct density dependence with lag year 1, derived from the population models described above, parameter *β*_1_) was normally distributed with some true, unobserved direct density dependence ($D_{s}$) and variance derived from the estimated sample standard deviation of the posterior mean from the first stage of population model fitting at each time series ($\sigma_{D.{\mathrm{est}}_{s}}^{2}$). The true unobserved direct density dependence was then modelled using a linear mixed model for each *i*th environmental variable (${\mathrm{env}}_{i,s}$) with normally distributed random error with variance ($\sigma_{D}^{2}$),

$$\begin{array}{l} D.{\mathrm{est}}_{s}\sim \text{normal}\left(D_{s},\sigma_{D.{\mathrm{est}}_{s}}^{2}\right),\\ D_{s}\sim \text{normal}\left(D.{\text{true}}_{s},\sigma_{D}^{2}\right),\\ D.{\text{true}}_{s}=\alpha +\beta_{i}\cdot {\mathrm{env}}_{i,s}. \end{array}$$

All models were fitted using R and JAGS using minimally informative priors for regression intercepts (α ~ normal(0, 0.0001)) and slopes (*β*_*i*_ ~ normal(0, 0.0001)), and for residual standard deviation (*σ*_*D*,*s*_ ~ uniform[0,1]). Models were fitted and convergence assessed as described above for the population models. We assessed variance explained by each model using *R* ^2^ following Nakagawa and Schielzeth (2013), calculating the ratio between the residual variance of the model of interest and the residual variance of the null model.

## 3 RESULTS

### 3.1 Population models

Posterior predictive checks demonstrated that in all species there was very good model fit, with most Bayesian *p* values close to 0.5 for all models and colonies (Supplementary Material S7). In just two species, Black Guillemot *Cepphus grylle* and Great Black‐backed Gull *Larus marinus*, there was some lack of fit at one of the breeding colonies for each species (Bayesian *p* value 0.095 for Black Guillemot, and 0.042 for Great Black‐backed Gull; Supplementary Material S7).

We found evidence for direct density dependence in eight of the ten species (model M1; Table 2), with only two species, Common Guillemot *Uria aalge* and Black Guillemot, showing greatest support for the null model (model M0; Table 2; Common Guillemot: ΔDIC: 5.6 for model M1; Black Guillemot: ΔDIC: 8.4 for the model M1). Although Lesser Black‐backed Gull showed greater support in the data for the model including density dependence, there was similar support in the data for the null model, indicating density independence (ΔDIC 1.1; Table 2).

**TABLE 2:** Summary of alternative population model estimates in breeding seabirds (M0: no effect of density; M1: Effect of density in previous year). DIC presented to assess support for alternative models. Results shown for M1 models for the fraction of time‐series for each species where negative density dependence was detected (no time‐series exhibited positive density dependence).The DIC value for the model with the most support in the data is highlighted in bold.

Species	DIC model M0	DIC model M1	ΔDIC	Number of colonies with direct density dependence
Common guillemot *Uria aalge*	**5966.0**	5971.6	5.6	8/20
Northern fulmar *Fulmarus glacialis*	6709.4	**6673.2**	36.2	16/29
Black‐legged kittiwake *Rissa tridactyla*	9123.3	**9098.1**	25.2	11/36
Razorbill *Alca torda*	5061.2	**5043.2**	18.0	10/21
Lesser black‐backed gull *Larus fuscus*	2917.5	**2916.4**	1.1	4/12
Arctic tern *Sterna paradisaea*	2912.4	**2905.1**	7.3	5/9
Black guillemot *Cepphus grylle*	**2266.9**	2275.3	8.4	4/12
European shag *Gulosus aristotelis*	5518.4	**5483.1**	35.3	11/25
Great black‐backed gull *Larus marinus*	2695.1	**2671.5**	23.6	7/15
Common tern *Sterna hirundo*	6572.1	**6546.5**	25.6	11/20

Overall, strong evidence for direct negative density dependence (lag 1 year) was detected in about 45% (75/167) of the time series across the eight species for which population models showing evidence for density dependent effects received the greatest support (Table 2). Evidence was detected in time‐series that were both increasing, decreasing and relatively stable over the period of time examined (1986–2016) (Supplementary Material S8).

We estimated relevant density‐dependent driven variation in population growth rate for all populations. These estimates varied across species, with density dependent effects on population growth rate for a 10% increase in mean population size tending to be weakest in Common Guillemot (range of estimates for multiplicative change in growth rate: 0.98–1.00, Figure S4), Razorbill (range: 0.96–1.00; Figure S4), Northern Fulmar (range: 0.96–1.01) and Lesser Black‐backed Gull (range: 0.95–1.00; Figure S4). Six species showed larger multiplicative reductions in population growth rate with a 10% increase in population size, with the greatest relative reductions occurring in European Shag (range of estimates for multiplicative change in growth rate: European Shag: 0.92–0.99; Black‐legged Kittiwake: 0.93–1.04; Black guillemot: 0.95–0.99; Common Tern: 0.94–1.00; Arctic Tern: 0.95–0.99; Great Black‐Backed Gull: 0.94–1.00; Figure S4).

### 3.2 Correlation of direct density dependence with environmental variation

#### 3.2.1 Accentuating effect of temporal variation in climate

**FIGURE 1:**
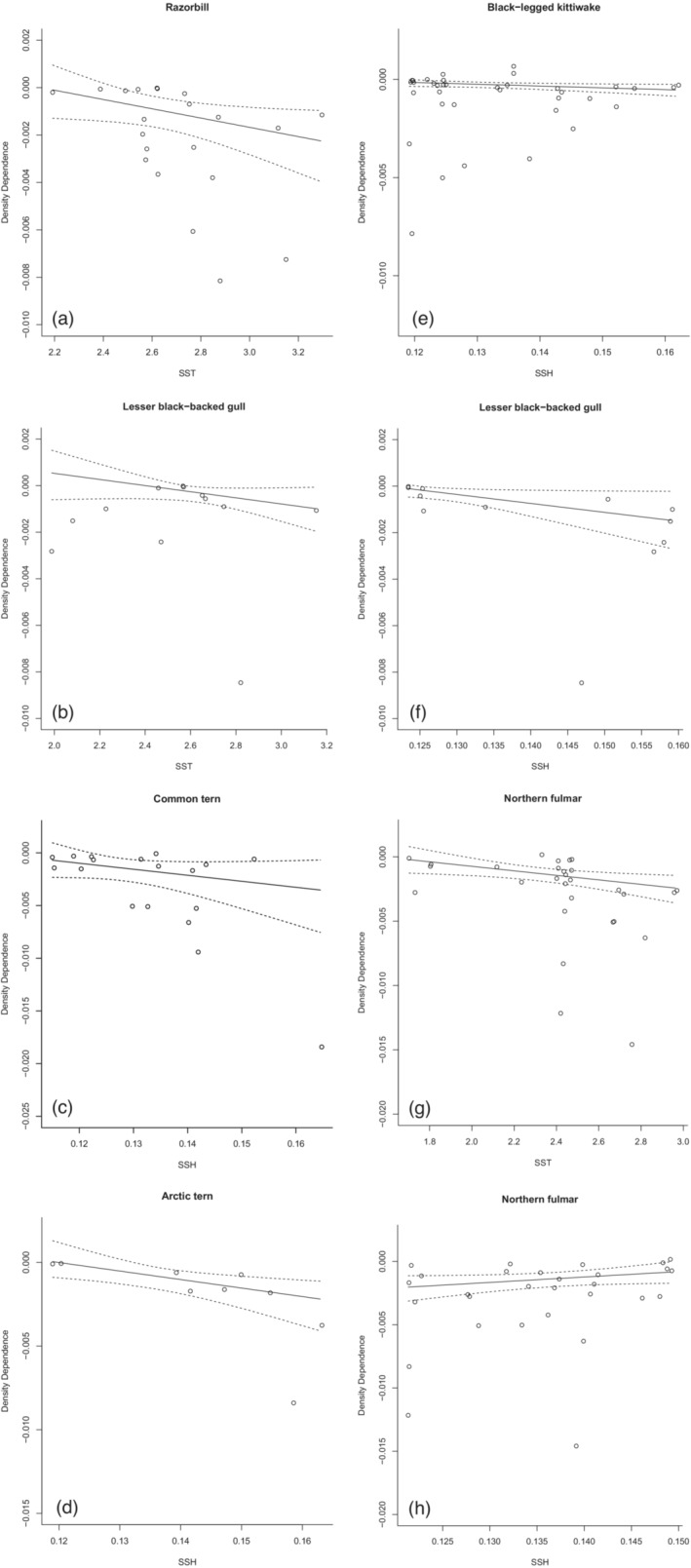
Relationship between strength of density dependence and temporal variation in sea surface temperature (SST) and sea surface height (SSH) over the breeding season for six seabird species for which strong evidence for correlative effects was detected (>90% posterior density was negative; in one species, Northern Fulmar, there was evidence for both increasing and decreasing density dependence with increasing temporal variation in climate. Solid lines are posterior means with 95% credible intervals (dotted lines). (a) Razorbill (SST), (b) Lesser Black‐backed Gull (SST), (c) Common Tern (SSH), (d) Arctic Tern (SSH), (e) Black‐legged Kittiwake (SSH); (f) Lesser Black‐backed Gull (SSH), (g) Northern Fulmar (SST), (h) Northern Fulmar (SSH).

Five of the ten species showed evidence for an accentuation of negative direct density dependence with increasing temporal variation in climate over both the entire year and the breeding season: Lesser Black‐backed Gull, Razorbill, Black‐legged Kittiwake, Arctic Tern and Common Tern (Table 3, Figure 1). In Lesser Black‐backed Gull, there was strong evidence that the strength of density dependence increased with both increasing variation in SST and SSH (Table 3, Figure 1). In Arctic Tern and Black‐legged Kittiwake, there was strong evidence that density dependence increased in strength with increasing variation in SSH, and slightly weaker evidence for this relationship in Common Tern (Table 3, Figure 1). Finally, in Razorbill, there was strong evidence for an increase in the strength of density dependence with increasing variation in SST (Table 3, Figure 1). One additional species, Northern Fulmar, showed evidence for increasing strength of density dependence with increasing temporal variation in climate when measured using SST, however in contrast, this species also showed evidence for a decrease in density dependence with increasing temporal variation in climate measured using SSH (Table 3, Figure 1). In four species, we detected no evidence for correlation between temporal variation in climate (SST or SSH) and the strength of direct density dependence: Great Black Backed Gull, Black Guillemot, Common Guillemot and European Shag (Table 3).

**TABLE 3:** Effect of environmental covariates on strength of density dependence on population growth rates for breeding UK seabirds. Environmental effects are grouped into temporal variation in climate (SST: Sea surface temperature; SSH: Sea surface height) and spatial variation in prey resources (CHL: Productivity; TF: Tidal fronts). Expected direction of relationship indicated in parentheses for each environmental covariate. Strong (>95% posterior density) and weak (>90% posterior density) evidence for the expected direction of the correlation denoted by ‘==’ and ‘=’, and by ‘≠ ≠ ’and ‘≠’ for evidence contrary to the expected direction of the correlation. For each effect, table includes posterior mean, 95% credible interval in parentheses, percentage of posterior distribution above or below zero, and model *R* ^2^.

	SST (−ve)	SSH (−ve)	CHL (+ve)	TF (+ve)
Northern fulmar	== −0.0017 (−0.0033, 0.00035) (99%) 0.09	*≠≠* 0.043 (−0.0065, 0.097) (96%) 0.05	−0.0042 (−0.023, 0.012) (67%) 0.03	0.020 (−0.030, 0.067) (80%) 0.0003
Lesser black‐backed gull	== −0.0025 (−0.005, 0.0002) (97%) 0.002	= −0.038 (−0.10, 0.011) (94%) 0.14	= 0.0045 (−0.0016, 0.013) (93%) 0.09	−0.012 (−0.048, 0.021) (76%) 0.001
Great black‐backed gull	0.000053 (−0.0049, 0.0045) (52%) 0.004	−0.015 (−0.19, 0.15) (57%) 0.002	*≠≠* −0.041 (−0.09, 0.0064) (96%) 0.12	0.036 (−0.031, 0.11) (88%) 0.13
Razorbill	== −0.0027 (−0.0057, −0.0002) (98%) 0.14	0.011 (−0.044, 0.069) (66%) 0.01	== 0.012 (−0.0022 0.027) (96%) 0.001	= 0.018 (−0.0097, 0.050) (91%) 0.03
Black‐legged kittiwake	0.00024 (−0.00024, 0.00075) (84%) 0.01	== −0.011 (−0.022, −0.0006) (98%) 0.02	= 0.0025 (−0.0010, 0.0059) (93%) 0.02	0.00049 (−0.0054, 0.0066) (57%) 0.01
Arctic tern	−0.00037 (−0.0036, 0.0026) (59%) 0.005	== −0.049 (−0.11, −0.017) (99%) 0.30	*≠* −0.016 (−0.050, 0.0074) (90%) 0.40	0.0050 (−0.028, 0.041) (65%) 0.02
Black guillemot	−0.0031 (−0.015, 0.0084) (70%) 0.01	0.014 (−0.088, 0.110) (62%) 0.06	−0.00059 (−0.0042, 0.0033) (64%) 0.07	−0.0064 (−0.049, 0.033) (62%) 0.13
Common guillemot	−0.00025 (−0.00073, 0.00015) (89%) 0.002	0.00068 (−0.0065, 0.0079) (57%) 0.004	0.00046 (−0.00081, 0.0019) (77%) 0.005	0.0023 (−0.0017, 0.0070) (88%) 0.01
Common tern	−0.00035 (−0.0016, 0.00071) (74%) 0.01	= −0.057 (−0.16, 0.019) (92%) 0.19	0.0019 (−0.025, 0.025) (59%) 0.02	0.0036 (−0.024, 0.035) (59%) 0.002
European shag	0.00043 (−0.0025, 0.0034) (62%) 0.003	−0.029 (−0.097, 0.026) (84%) 0.10	0.0043 (−0.011, 0.021) (71%) 0.04	−0.0095 (−0.043, 0.016) (73%) 0.07

#### 3.2.2 Mitigating effect of spatiotemporal variation in resources

**FIGURE 2:**
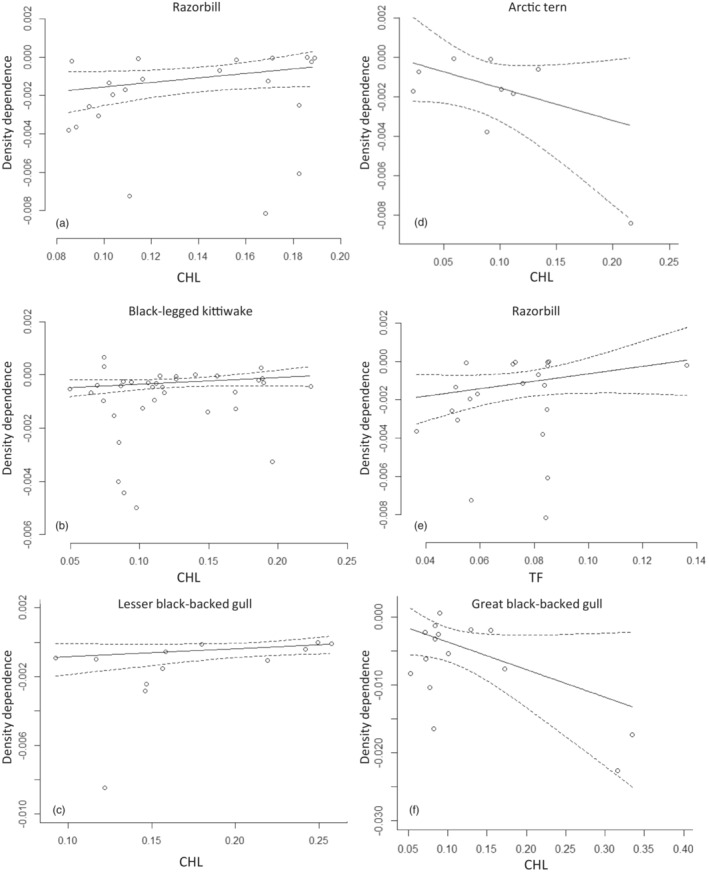
Relationship between strength of density dependence and spatiotemporal variation in chlorophyll (CHL) and tidal fronts (TF) over the breeding season for five seabird species for which strong evidence for correlative effects was detected (>90% posterior density was positive in three species, and in two species, Arctic tern and great black‐backed gull, >90% posterior density was negative). Solid lines are posterior means with 95% credible intervals (dotted lines). (a) Razorbill (CHL); (b) Black‐legged kittiwake (CHL); (c) Lesser black‐backed gull (CHL); (d): Arctic tern (CHL), (e): Razorbill (TF), (f) Great black‐backed gull (CHL).

We detected strong evidence for the anticipated weakening of negative density dependence with increasing spatiotemporal variation in resources in three species; Razorbill, Lesser Black‐backed Gull and Black‐legged Kittiwake (Table 3, Figure 2). In contrast to the hypothesized effect, we found strong evidence for an increase in the strength of negative density dependence with increasing spatiotemporal variation in two species, Great Black‐backed Gull and Arctic Tern (Table 3; Figure 2).

In the remaining five species, Northern Fulmar, Black Guillemot, Common Guillemot, Common Tern and European Shag, we detected no evidence for a link between spatiotemporal variation in prey resources and the strength of direct density dependence (Table 3).

Variance explained by the models testing effects of temporal variation in climate and spatial variation in resources was generally low (Table 3). In species for which strong evidence of effects was detected, variance explained ranged from <1% to 40%, and in other species it spanned <1% to 13% (Table 3).

## 4 DISCUSSION

Assessing the intensifying threats of environmental change and increasing anthropogenic pressures to ecological systems requires broad multi‐species analyses to unravel the fundamental processes whereby environmental variation affects population dynamics within communities. Although density dependence is widespread amongst seabirds including those in North West Europe, the regulatory processes are highly context dependent (Horswill et al., 2017). Here, we present a multispecies test for how members of the UK seabird community respond to two differing sources of environmental variation—temporal variation in climate and spatiotemporal variation in prey resources—operating via opposing effects on density dependence in population growth rates. Our results provide new empirical evidence that the interaction between differing patterns of environmental variation and strength of population regulation in these marine top predators closely resembles the interplay previously demonstrated in populations of terrestrial mid‐trophic herbivores. These findings extend an emerging paradigm into marine species and higher trophic levels, thereby lending important weight to the role of these processes in shaping animal population regulation more widely.

Although density dependent effects in marine birds appear pervasive, the underlying mechanisms remain poorly understood (Fay et al., 2017; but see Barbraud et al., 2018; Cury et al., 2011; Goyert et al., 2017). A recent review of evidence for density dependence in demographic rates of 31 species of seabirds in North West Europe identified significant effects in 88% of studies covering 23 species (Horswill et al., 2017). We found strong evidence for direct negative density dependence in 45% of the seabird abundance time series examined, with evidence occurring in eight out of the ten species. Direct negative density dependence was most frequently identified in Arctic Tern, Northern Fulmar, Common Tern, Razorbill and Great Black‐backed Gull, with around 50% of time series for these species demonstrating strong evidence. European Shag and Common Guillemot time series provided strong evidence in around 40% of time series, with around 30% of time series exhibiting strong evidence for Lesser Black‐backed Gull, Black Guillemot and Black‐legged Kittiwake.

The strength of direct negative density dependence varied markedly across species and abundance time series, but in general, the strongest effects in relation to a 10% increase in mean population size, occurred in European Shag where the population growth rate was estimated to decrease by as much as 10% in some of the time series. The strength of density dependence was also relatively large in several time series for Black‐legged Kittiwake (up to 7% reduction in some cases), Great Black‐backed Gull (up to a 6% decrease), and Arctic Tern and Black Guillemot (up to a 5% decrease). Estimates for the effect of density dependence on population growth rate were weakest in Common Guillemot (maximum 2% decrease in population growth rate) and Razorbill (maximum 4% decrease). It is plausible that the need for a correction factor to convert counts of individuals to pairs compromised our ability to detect strong density dependence in Common Guillemot and Razorbill. There is inherent uncertainty in estimating this conversion factor compounded by applying a value derived at one location (the Isle of May in eastern Scotland) to other populations where attendance patterns may be different (Supplementary Material S2). The additional noise that arises from these sources of uncertainty is likely to lead to reduced statistical power for detecting density dependence. More generally, across all species, it is possible that some populations may experience density dependent effects via more resolved mechanisms such as delayed recruitment of pre‐breeders, but that due of buffering of other vital rates such as survival, these effects may be masked when only considering density dependent regulation of population growth rates.

### 4.1 Drivers of density dependence

We tested if the strength of density dependence in population growth rates in ten seabird species was stronger in populations experiencing greater temporal variation in climate, and weaker in populations subjected to higher spatiotemporal variation in food resources. We found more corroborating evidence in support of an accentuation of density dependence with increasing temporal variation in climate (in five of the ten species, with an additional species showing mixed support, both for and against; Table 4), than for a weakening of density dependence with increasing spatiotemporal variation in resources (in three of the species, with two species showing contrary results; Table 4). In a further three species, Black Guillemot, Common Guillemot and European Shag, we detected no relationships between strength of density dependence and our environmental variables.

**TABLE 4:** Summary of the interplay between the strength of density dependence and environmental variation in ten species of UK seabirds. Anticipated effects include a strengthening of density dependence with increasing variation in climate, and a weakening of density dependence with increasing spatiotemporal variation in prey resources. Grey columns indicate species showing opposite responses to the anticipated effects. No effects were detected for black guillemot, common guillemot or European shag.

Temporal variation in climate		Spatiotemporal variation in resources	
Strengthened	Weakened	Strengthened	Weakened
Lesser black‐backed gull	Northern Fulmar	Great black‐backed gull	Lesser black‐backed gull
Razorbill		Arctic tern	Razorbill
Black‐legged Kittiwake			Black‐legged Kittiwake
Arctic Tern			
Common Tern			
Northern Fulmar			

The dependence of the effect of density on variation in climate likely arises because extremes of climate act to temporarily reduce the carrying capacity of the local environment by increasing the energetic requirements of animals, moving them away from their physiological optimum, and/or by reducing food availability (Wang et al., 2006). These effects are manifest as an intensification of feedbacks from population density to individual survival and breeding success. Negative effects of population density on survival have previously been reported in some seabird species, and have been linked with the influence of adverse climatic conditions increasing at higher population densities (Barbraud & Weimerskirch, 2003; Frederiksen & Bregnballe, 2000; Horswill et al., 2017). More recently, the carrying capacities of five seabird species in Alaska have been shown to vary systematically with climate over four decades, linked to deteriorating prey availability (Goyert et al., 2017). In our study, it is likely that breeding populations in areas exposed to greater temporal variation in climate, both in terms of sea temperature and sea surface height, suffer from more acute temporary reductions in carrying capacity than populations in places with a more stable climate. Increased climatic variation would exacerbate the effects of density on processes affecting population growth, such as resource limitation of suitable nesting sites (forcing individuals to use poorer quality nest sites, potentially more exposed to weather) and food (via climate‐mediated impacts on prey), and by increasing the energetic requirements of seabirds, potentially aggravating competition between individuals. Population growth rates of UK seabirds have been reported to be limited by terrestrial and marine factors including the number of good quality breeding sites in Black‐legged Kittiwakes and Common Guillemots (Bennett et al., 2022; Coulson, 1983; Kokko et al., 2004; Porter & Coulson, 1987), by density dependent depletion of prey in Northern Gannets (Davies et al., 2013; Lewis et al., 2001), by resource limitation affecting recruitment in Common Guillemots (Crespin et al., 2006), and by territory formation under high population densities in Herring Gulls *Larus argentatus* (Coulson et al., 1982; Raven & Coulson, 1997). More widely, density dependence in seabirds beyond UK waters is affected by a number of drivers, in relation to food resources for three seabird species in the northern Humboldt Current System off the coast of Peru (Barbraud et al., 2018), in Antarctic species (e.g., Pacoureau et al., 2019), and in a gull species in the western Mediterranean (Genovart et al., 2018), and in relation to limited breeding sites in Antarctic seabird species (e.g., Southwell & Emmerson, 2020).

The proposed mechanism for the mediating effect of spatiotemporal variation on density dependence is through facilitating selective foraging by consumers allowing individuals to cope with food resources in which nutrient and energetic concentrations vary over time, and to buffer against food shortages in times of adversity. This mechanism is likely to be particularly strong in highly mobile foragers such as seabirds that may travel more than 200 km in a single foraging trip (Thaxter et al., 2012). The seabird species considered in our study primarily forage on younger age classes of forage fish, meaning there is the potential for considerable spatiotemporal variation in the energy content of available prey over time due to both prey phenology and maturation rates, and to spatial structuring in forage fish populations (MacDonald et al., 2015; Rindorf et al., 2000). Reductions in spatiotemporal variation in these key prey resources can restrict the range of available prey to foragers, and this compression may hamper the ability of mobile foragers to respond to temporal variation in prey quality through selective use of space (Wang et al., 2006). Direct studies of this mechanism in seabirds are rare. However, three species of seabirds breeding in the southeastern Bering Sea shifted their dietary niche in response to changing environmental conditions, measured using sea temperatures, and this shift was mediated by their ability to access spatially heterogeneous foraging habitats (Kokubun et al., 2018; Will & Kitaysky, 2018). Foraging habitat variation does, therefore, appear to be a valid mechanism allowing breeding seabirds to exploit changing prey availability in relation to climatically driven oceanographic changes, and to potentially buffer against periods of low food availability through access to diverse habitats and spatial variation in forage resources.

The Great Black‐backed Gull was one of only two species to show strong evidence for contrary relationships to those expected, exhibiting a strengthening of density dependence with increasing spatiotemporal variation in prey resources (using Chlorophyll A concentration as a proxy for resource availability). A similar, but weaker effect was also detected in Arctic Tern. Great Black‐backed Gulls are dietary generalists. Thus in contrast to the other study species that predominantly feed on forage fish or invertebrates, Great Black‐backed Gulls also predate and scavenge a wide variety of nonpiscivorous prey including other seabirds and marine and terrestrial mammals (Schreiber & Burger, 2001). This suggests that the processes linking environmental variation and density dependence in generalist top predators, feeding partly on other predatory species, are more complex and may well fail to be adequately captured using the metrics tested here.

Broadly across the study species, our models for effects of environmental variation on density dependence explained a limited amount of the observed variation (ranging from <1% to 40% across models for the seven species in which strong effects were detected), despite identifying strong relationships between climate variables and strength of density dependence. This is not uncommon with ecological data, particularly when there is uncertainty around the strength of density dependence arising from initial population models. Moreover, seabird population processes will respond to environmental fluctuations from year to year, including lag effects, which are difficult to capture precisely with environmental variables that have relatively coarse spatial and temporal resolution. These limitations engender caution when interpreting results for the effects of environmental variation on the strength of density dependence. However, our findings add to the growing body of evidence for these types of effects, suggesting important qualitative support for these underlying processes of population regulation in both terrestrial and marine environments.

### 4.2 Future work

Our results are based upon models for density dependence fitted to point estimates of counts for breeding birds at colonies. However, many populations of marine birds have large non‐breeding populations that are often difficult to quantify but likely to strongly influence the strength of density dependence through con‐specific competition for resources (Horswill et al., 2017). The potential effect of nonbreeders on population growth rates is limited in the context of our population models to those birds that are physiologically capable of breeding, but are currently not breeding due to skipping a year or failing to establish a pair bond or nest site. There is evidence to suggest that in some species, such as Common Guillemots, the proportion of nonbreeding adults is not significantly affected by population size (Crespin et al., 2006; Reed et al., 2015). If this also applies to other species in our analysis, then we would not expect the evidence for density dependent effects to have been biased by changing proportions of non‐breeders over time. However, future research should test this assumption and estimate if the proportion of non‐breeding adults varies in relation to environmental conditions, and how this may affect key population processes such as productivity, dispersal and density dependent effects.

Evidence for an attenuation of density dependence with increasing spatiotemporal variation in resources tended to be less strong than that for a strengthening of density dependence with increasing temporal variation in climate. This may in part be due to our use of proxies for spatiotemporal variation in prey (related to productivity and oceanography), due to the lack of direct data on forage fish abundance at appropriate temporal and spatial scales. Although previous work has demonstrated strong links between seabird behaviour and demography in relation to these proxies (Tremblay et al., 2009; Waggitt et al., 2018), they are, nonetheless, several steps removed from the real prey landscape to which the seabirds are responding. It is, therefore, not unexpected that evidence of effects associated with spatiotemporal variation in prey proved to be harder to detect in our analysis. More in depth analyses of density dependence in seabird populations utilising direct measures of spatiotemporal variation in forage fish prey should elucidate whether seabird population dynamics are more strongly influenced by spatiotemporal variation in food resources or by temporal variation in climate.

## 5 CONCLUSIONS

Our findings demonstrate multispecies support for an emerging theory for how different sources of environmental variation may consistently shape the dynamics and regulation of animal populations in mobile upper‐trophic marine species such as seabirds. The weight of evidence in our study supported a general multi‐species effect of accentuated density dependence with increasing temporal variation in climate, with lesser support for an amelioration of density dependence with increasing spatiotemporal variation in resources. These results highlight the need for empirical studies to elucidate the underpinning mechanisms whereby spatiotemporal variation in resources may allow mobile foragers to buffer against resource scarcity and environmental stressors such as variation in extreme weather. Undertaking such studies is particularly challenging in the marine environment, where foraging animals may range over hundreds, sometimes thousands, of kilometres in a single foraging trip, and estimates for the distribution and abundance of forage fish are temporally and spatially sparse. However, the existence of such mechanisms, as suggested by our results, offers significant potential benefits for marine organisms faced with predicted increases in extreme climatic events, increasing impacts of long‐term climate change and other anthropogenic impacts such as overfishing, plastic pollution and offshore renewable developments. If marine protected areas are able to encompass areas of spatiotemporal variation in key prey resources at an appropriate scale to marine predators their effectiveness is likely to be enhanced (Oppel et al., 2018). Many seabird species are recognised as indicators of ecosystem status (Cook et al., 2014; Sydeman et al., 2017; Tam et al., 2017) and used as barometers for assessing the health of marine ecosystems under statutory obligations in many countries (e.g. the EU Marine Strategy Framework Directive). Eliciting understanding of the relationships between environmental variation and key demographic processes in this community is, therefore, of prime importance to efforts to safeguard our oceans from the myriad of threats they currently face.

## AUTHOR CONTRIBUTIONS

Kate R. SearleAdam Butler, N. Thompson Hobbs, Francis Daunt and Sarah Wanless conceived the ideas and designed the methodology; Kate R. Searle and Adam Butler analysed the data with contributions from James J. Waggitt, Peter G. H. Evans and Maria I. Bogdanova; Kate R. Searle led the writing of the manuscript, supported by Francis Daunt and Sarah Wanless. All authors contributed critically to the drafts and gave final approval for publication.

## CONFLICT OF INTEREST

There are no conflicts of interest to declare for any of the authors of this manuscript.

## DATA AVAILABILIY STATEMENT

Data are available from the Seabird Monitoring Programme (SMP) repository, run by the British Trust for Ornithology: https://app.bto.org/seabirds/public/index.jsp (Mitchell et al., 2004). To acquire the data used in our analysis from the SMP, request seabird colony count data covering the species and years of interest detailed in our methods section, which will then be made available for download, allowing replication of our analysis.

## Supporting information


Appendix S1
