# Supplementary material for: Opposing effects of spatiotemporal variation in resources and temporal variation in climate on density dependent population growth in seabirds
Source: J Anim Ecol. 2022 Oct 31;91(12):2384–99. doi: 10.1111/1365-2656.13819 (PMC10092667; doi:10.1111/1365-2656.13819)
Supplement: Supplementary file 1 — Appendix S1 [file JANE-91-2384-s001.docx]

**Supplementary Material**

**S1. A:** Details for time series dropped (Seabird Monitoring Programme, SMP, “sub-sites”) based on removing sites where the maximum observed count over the entire time series was less than the 10% sample quantile of the maximum counts over all time series for that species. **B**: Summary of data on length of time series used in population modelling for each species, showing maximum length (years), mean length (years), and standard deviation (sd: years).

| **A** |  |  |  |
| --- | --- | --- | --- |
| **Species** | **10% sample quantile of maximum count across all time series** | **Number of sites dropped** | **SMP sub-site name(s) with maximum count in parentheses** |
| Arctic tern | 152.4 | 1 | Ballantrae (35) |
| Black guillemot | 114.8 | 1 | St Bees Head – Tysties (15) |
| Common guillemot | 218.2 | 3 | Caldey Island (38)  Mewsford Arches to Crickmail Point (149)  The Castle (43) |
| Common tern | 57.2 | 4 | Abberton Reservoir (44)  Hanningfield Reservoir (26)  Newtown NNR (15)  Rye Meads RSPB (52) |
| Northern fulmar | 27.4 | 4 | Flimston Bay to Mewsford Arches (11)  New Quay to Trevallen (19)  St Govan's Chapel to New Quay (11)  St Margaret's Island (18) |
| Great black-backed gull | 18.4 | 6 | An Glas-eilean (13)  Caldey Island (11)  Eilean Gainimh (17)  Eilean Mhic Neill (12)  Green Bridge of Wales to Flimston Bay (Elegug Stacks) (4)  Sands of Forvie (9) |
| Black legged kittiwake | 197.2 | 0 |  |
| Lesser black-backed gull | 25.0 | 1 | Saddle Point to Griffith Lorts Hole (Stackpole Head) (20) |
| Razorbill | 44.8 | 3 | Boulby Cliffs (28)  Mewsford Arches (32)  The Castle (38) |
| European shag | 37.4 | 5 | Caldey Island (5)  Green Bridge of Wales to Flimston Bay (Elegug Stacks) (8)  New Quay to Trevallen (2)  Saddle Point to Griffith Lorts Hole (Stackpole Head) (6)  St Margaret's Island (25) |
| **B** |  |  |  |
| **Species** | **Maximum number of years** | **Mean number of years** | **Standard deviation of number of years** |
| Arctic tern | 46 | 39.2 | 7.2 |
| Black guillemot | 31 | 27.5 | 1.6 |
| Common guillemot | 30 | 28.3 | 2.1 |
| Common tern | 46 | 41 | 7.2 |
| Northern fulmar | 30 | 28.9 | 0.9 |
| Great black-backed gull | 30 | 28.1 | 2.1 |
| Black legged kittiwake | 30 | 27.8 | 2.5 |
| Lesser black-backed gull | 30 | 28.1 | 2.4 |
| Razorbill | 30 | 27.4 | 3.6 |
| European shag | 30 | 27.4 | 2.6 |

**S2. Methods and results for data model used to convert observations of individuals to breeding pairs for two species (Common guillemot and Razorbill)**

For three species (Common guillemot, Black guillemot and Razorbill) the count unit was for breeding abundance at colonies in units of individual birds rather than breeding pairs (see Walsh et al. 1995 for full details). For two of these species, Common guillemot and Razorbill, Harris et al. (2015a,b) estimated a species-specific yearly value of the parameter, ‘*k*’, which we used to convert from individuals to breeding pairs, using data from breeding populations on the Isle of May NNR in the Firth of Forth, Scotland, over the period 1986-2016. Estimates for this adjustment have been made at a handful of other colonies for these two species (Shetland; Harris et al. 2015a). However, because other estimates do not cover the entire time period used in our analyses and are unavailable at most breeding colonies, we chose to use the annual values of ‘*k*’ derived from the Isle of May NNR, thereby assuming temporal changes in this parameter have been similar across all colonies for these species. The annual values of the adjustment parameter at the Isle of May were smoothed for each species, in order to remove the effects of year-to-year sampling variation, using generalized additive models (GAMs). The GAMs were fitted using the “gam” function in the mgcv package in R (Wood, 2011), assuming a smooth trend with “year”, and the ‘predict.gam’ function was then used to generate the estimated mean $m_{kt}$ and standard error $s_{kt}$ of the predicted *k*-value for each species in each year of observation $t$. Yearly-specific estimates for the adjustment for Black guillemots were unavailable, therefore this species was modelled as counts of individuals.


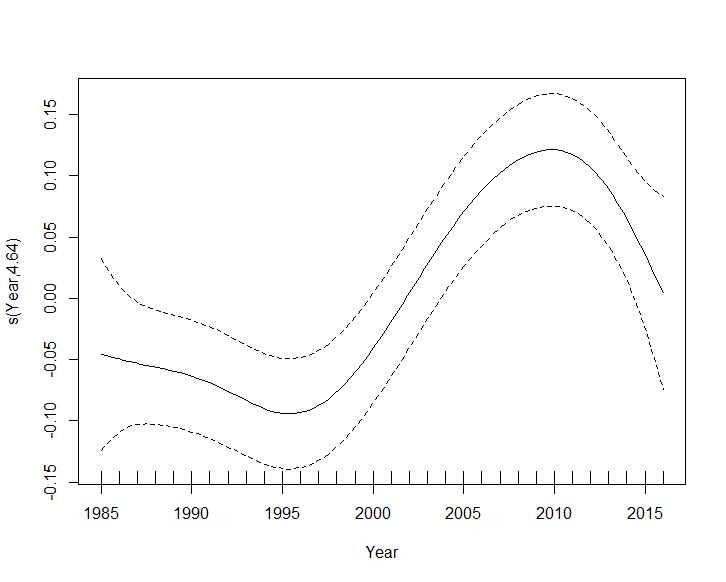
For Common guillemots the GAM explained 65% of the variation in estimated *k* values (intercept: 0.75, SE:0.010, t:72.19, P <0.001; smooth term edf: 4.64, Ref.df: 5.69, F: 10.09, P <0.001), and the model indicated that for this species *k* values have increased from the mid-1990s to 2010, with a subsequent decline (Fig. S2a).

*Fig S2a. Predicted GAM k-values for common guillemots at IOM during 1985-2016. The dotted lines represent the 95% confidence interval.*


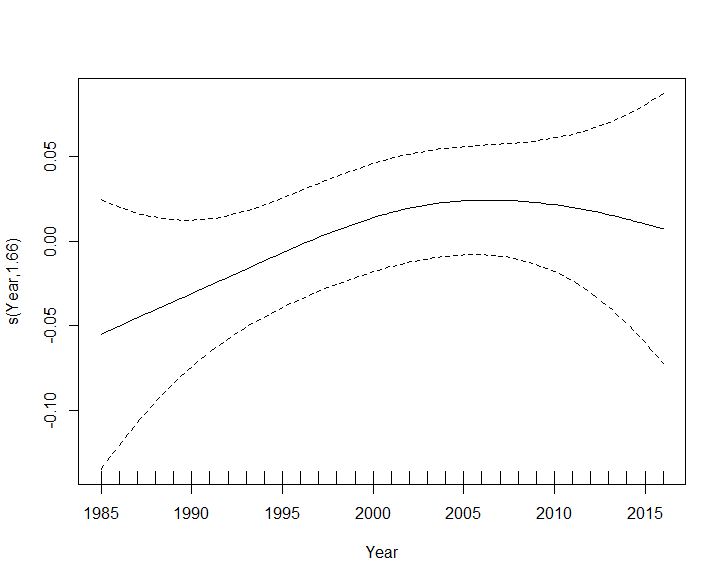
For Razorbills the GAM performed more poorly, explaining only 5% of the variation in estimated *k* values (intercept: 0.80, SE:0.018, t: 44.93, P <0.001; smooth term edf: 1.66, Ref.df: 2.06, F: 1.12, P 0.32), and the model indicated that for this species *k* values have gradually increased from 1985 with a slight decline from the mid-2000s (Fig. S2b).

*Fig S2b. Predicted GAM k-values for razorbills at IOM during 1985-2016. The dotted lines represent the 95% confidence interval.*

*References for S2*

*Harris, M. P., Heubeck, M., Newell, M. A. & Wanless, S. (2015a) The need for year-specific correction factors (k values) when converting counts of individual Common Guillemots Uria aalge to breeding pairs. Bird Study 62: 276–279.*

*Harris, M.P., Newell, M.A. & Wanless, S. (2015b) The use of k values to convert counts of individual Razorbills Alca torda to breeding pairs. Seabird 28: 30–36.*

**Table S3. Maximum clutch sizes used in setting minimally informative prior for the per capita intrinsic rate of population increase (**$\beta_{0s}$**) in each population model for each species. Respective lower (min) and upper (max) bounds for prior are shown for each species.**

| **SPECIES** | **CLUTCH SIZE** | **Source** | $\beta_{0s}\text{min}$ |  |
| --- | --- | --- | --- | --- |
| Common guillemot | 1 | Brooks & Perrins (1996) | -1.61 | 0.41 |
| Northern fulmar | 1 | Schreiber & Burger (2001) | -1.61 | 0.41 |
| Black-legged kittiwake | 3 | Schreiber & Burger (2001) | -1.61 | 0.92 |
| Razorbill | 1 | Brooks & Perrins (1996) | -1.61 | 0.41 |
| Lesser black-backed gull | 3 | Schreiber & Burger (2001) | -1.61 | 0.92 |
| Arctic tern | 2 | Schreiber & Burger (2001) | -1.61 | 0.69 |
| Black guillemot | 2 | Schreiber & Burger (2001) | -1.61 | 0.69 |
| European shag | 4 | Schreiber & Burger (2001), Brooks & Perrins (1996) | -1.61 | 1.10 |
| Great black-backed gull | 3 | Schreiber & Burger (2001) | -1.61 | 0.92 |
| Common tern | 4 | Schreiber & Burger (2001) | -1.61 | 1.10 |

**S4. Estimates for the strength of density dependence across all species and sites**


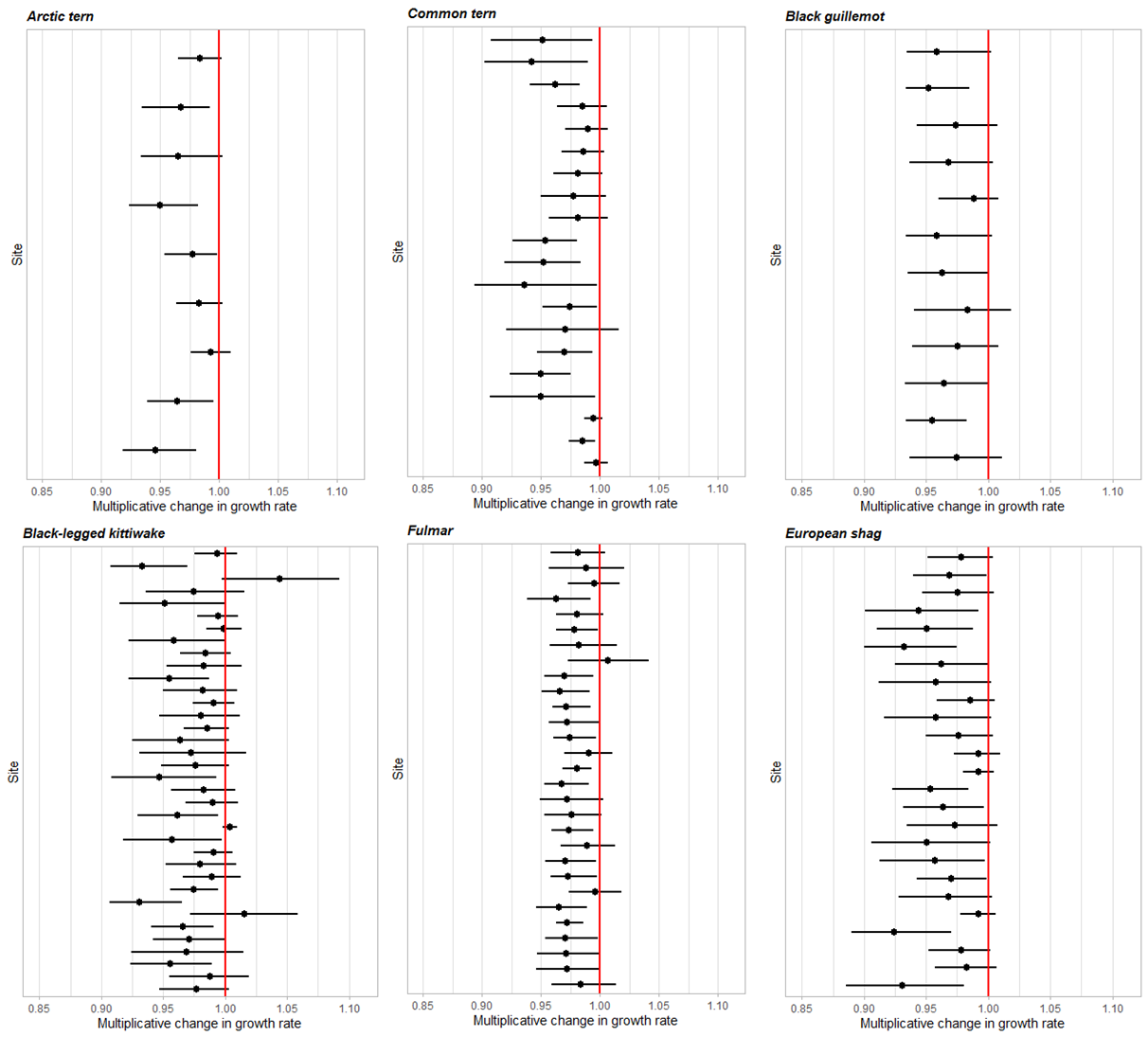


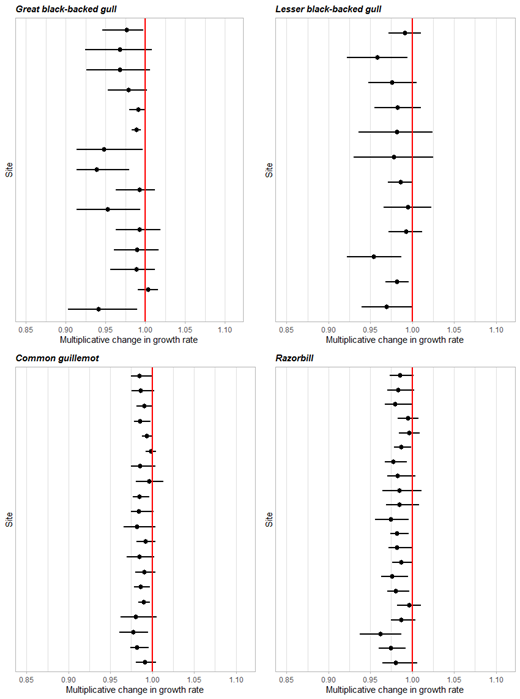


**Figure S4**. Estimates for the strength of density dependence, with a lag of one year, for each population modelled for ten species of seabirds breeding in the UK. For each species, the plot shows the posterior mean estimates (solid circles) and 95% credible intervals (horizontal bars) for the strength of lag 1 year density dependence at each site. The strength of density dependence is presented as the multiplicative proportional change in population growth rate, based upon the expected change in growth rate arising from a 10% increase in mean observed population size, *Nmean_t_*,, derived as (exp(0.1**Nmean_t_**β_1_)) where β_1_ is the lag 1 posterior mean from the fitted population model. Note that for Common guillemot and Razorbill the mean estimated adjustment factor from 1981-2016 was used to convert raw counts to breeding pairs (Common guillemot: 0.74; Razorbill: 0.80). Density dependent effects with strong support in the data are those in which the 95% credible interval for the multiplicative change does not include a value of one (i.e., a value of one indicates no change in population growth rate due to density dependence, shown with a vertical red line).

**S5. Full mathematical expression for posterior and fully factored joint distribution**

The posterior and factored joint distribution for the *j*^th^ species are

$$\left[ \boldsymbol{N}_{j,},\boldsymbol{K}, \beta,\sigma_{\epsilon}^{2}|y_{j} \right]\propto\prod_{t\in w_{j}} \left[ y_{j,t}|N_{j,t},K_{t} \right]\left[ N_{j,t}|\mu_{j,t} ,\sigma_{\varepsilon}^{2} \right]\left[ K_{t}|k_{mt},k_{st} \right]\left[ \beta\right]\left[ \sigma_{\varepsilon}^{2} \right]$$

$$y_{j,t}\sim\text{Poisson}\left( N_{j,t}K_{t} \right)$$

$$K_{t}\sim\text{normal}\left( k_{mt},k_{st}^{2} \right)$$

$$N_{j,t}\sim\text{lognormal}\left( \mu_{j,t},\sigma_{\varepsilon}^{2} \right)$$

$$\mu_{j,t}=\log\left( N_{t-1} \right)+\beta_{0}+\beta_{1}N_{t-1}+\beta_{2}N_{t-2}$$

$$\beta_{0}\sim\text{uniform}\left( -1.61,\log\left( 1+\frac{c_{j}}{2} \right) \right)c_{j}=\text{maximum clutch size}$$

$$\beta_{1}\sim\text{normal}\left( 0,10000 \right)$$

$$\beta_{2}\sim\text{normal}\left( 0,10000 \right)$$

$$\sigma_{\varepsilon}\sim\text{uniform}\left( 0,2 \right)$$

Where $w_{j}$ is a set indexing years of non-missing data for species *j*. Parameters $k_{mt}$ and $k_{st}$ are the annual mean and standard deviation of the *k*-values for adjusting counts of individuals to breeding pairs in two species (common guillemot and razorbill), derived from the Isle of May data (Supplementary Material S2). The above represents model M2; model M1 is a special case of model M2 where $\beta_{2}$ = 0, and model M0 is a special case of model M2 where both $\beta_{1}$ and $\beta_{2}$ = 0.

**S6. Spatial pattern for environmental variables describing temporal variation in climate and spatial variation in resources in UK waters.**

Temporal variation in climate was described using Sea Surface Height (SSH) or Sea Surface Temperature (SST) calculated over the breeding season. Spatial variation in resources was described using productivity (CHL) or tidal fronts (TF) calculated over the breeding season. The most temporally variable regions for both SSH and SST were in the eastern North Sea along the continental coast, with additional high variability around the north and west coast of Scotland (SSH) and the English Channel (SST) (S6a). For spatial variation in productivity (CHL), the greatest variation occurred in the North Sea, along inshore areas of the western coast of the UK, and off the shelf edge off western Scotland and Ireland (Fig. S6b). Spatial variation in tidal fronts was quite fine scale, with areas of high variability occurring throughout the North Sea, in northern waters up around Shetland, Orkney and Iceland, and along the western edge of the UK (Fig. S6b).


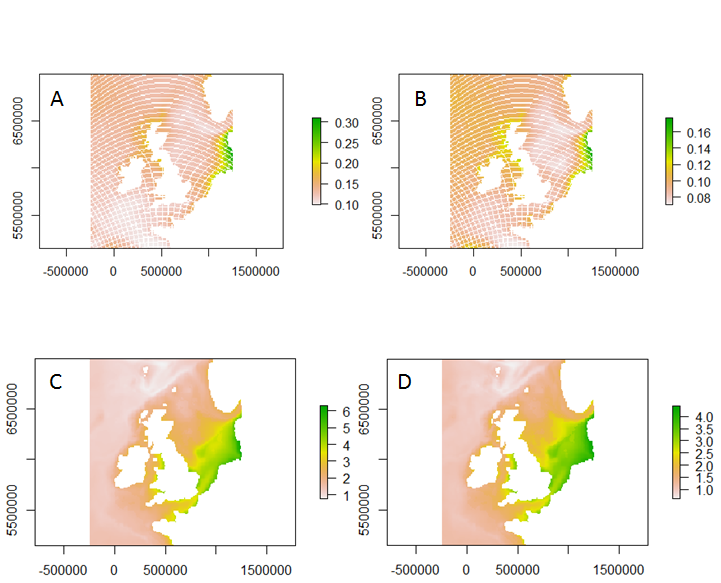


**Figure S6a.** Spatial pattern in environmental variables used to describe temporal variation in climate in UK waters. Upper panel: temporal variation in SSH calculated over the breeding season. Lower panel: temporal variation in SST calculated over the breeding season.


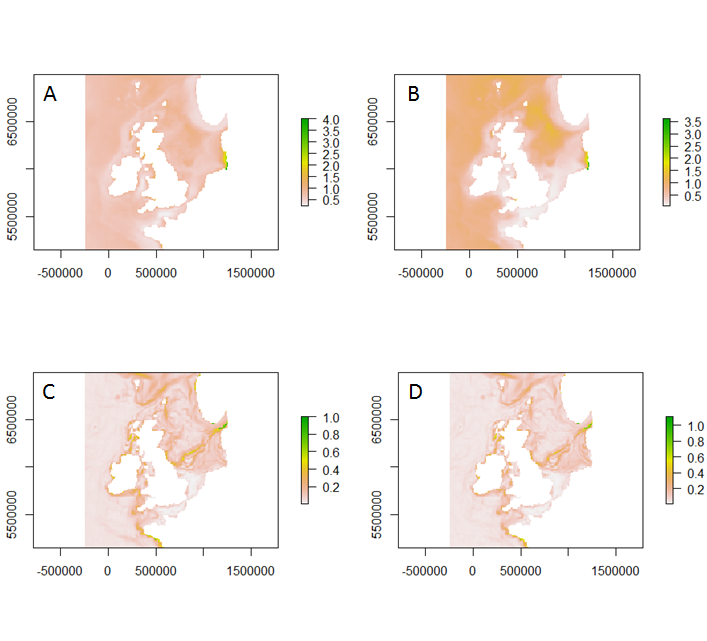
**Figure S6b.** Spatial pattern in environmental variables used to describe spatial variation in resources in UK waters. Upper panel: spatial variation in CHL calculated over the breeding season. Lower panel: spatial variation in TF calculated over the breeding season.

**Table S6. Foraging range for each species used to define the at-sea area around each breeding colony over which to create environmental variables for temporal variation in climate and spatial variation in resources. Months used to define breeding season for each species also shown.**

| **SPECIES** | **Mean maximum reported foraging range (km)** | **Spatial range used for environmental covariates (km)** | **Reference** | **Breeding season months** |
| --- | --- | --- | --- | --- |
| Common guillemot | 84.2 (135) | 150 | Wakefield et al. 2017 | April - July |
| Northern fulmar | 400 (580) | 250 | Thaxter et al. 2012 | April - August |
| Black-legged kittiwake | 60 (120) | 100 | Wakefield et al. 2017 | April – August |
| Razorbill | 48.5 (95) | 100 | Wakefield et al. 2017 | April – July |
| Lesser black-backed gull | 141 (181) | 150 | Thaxter et al. 2012 | April - July |
| Arctic tern | 24.2 (30) | 30 | Thaxter et al. 2012 | May - July |
| Black guillemot | 26.8k (single report) | 30 | Thaxter et al. 2012 | April - August |
| European shag | 14.5 (17) | 30 | Wakefield et al. 2017 | March - August |
| Great black-backed gull | 20 | 30 | Thaxter et al. 2012 | April - July |
| Common tern | 15.2 (30) | 30 | Thaxter et al. 2012 | May - July |

*References for species foraging ranges:*

Brooks, D. & C. Perrins (Eds). 1996. Birds of the Western Palearctic. Oxford University Press.

Schreiber, E. A. & J. Burger (Eds). 2001. Biology of Marine Birds. CRC Press, Marine Biology Series.

Thaxter, C. B., B. Lascelles, K. Sugar, A. Cook, S. Roos, M. Bolton, R. H. W. Langston, and N. H. K. Burton. 2012. Seabird foraging ranges as a preliminary tool for identifying candidate Marine Protected Areas. Biological Conservation **156**:53-61.

Wakefield, E. D., E. Owen, J. Baer, M. J. Carroll, F. Daunt, S. G. Dodd, J. A. Green, T. Guilford, R. A. Mavor, P. I. Miller, M. A. Newell, S. F. Newton, G. S. Robertson, A. Shoji, L. M. Soanes, S. C. Votier, S. Wanless, and M. Bolton. 2017. Breeding density, fine-scale tracking, and large-scale modeling reveal the regional distribution of four seabird species. Ecological Applications **27**:2074-2091.

**S7 Posterior predictive checks for best-fitting population model for all species.**

**Table S7** Bayesian P values arising from posterior predictive checks for each of the best fitting population models per species and breeding population. Population models denoted as follows: direct density dependence of lag 1 year only (M1); direct and delayed density dependence of lag 1 and 2 years (M2); no density dependence (M0). Bayesian P values are used to test whether the model fitted could have given rise to the observed data; values close to 0.5 indicate strong support for the model being appropriate for the data; values of less than 0.1 or greater than 0.9 suggest the model is a poor fit and unlikely to have given rise to the observed data. Problematic values are highlighted in grey. AT: Arctic tern; BG: Black guillemot; CT: Common tern; CG: Common guillemot; RZ: Razorbill; FUL: Northern fulmar; GBB: Great black-backed gull; KW: Black-legged kittiwake; LBB: Lesser black-backed gull; SH: European shag.

| site | AT  (M2) | site | BG  (M 0) | site | CT  (M2) | Site | CG  (M0) | Site | RZ (M1) | site | FUL  (M2) | site | GBB  (M2) | site | KW  (M1) | site | LBB  (M1) | site | SH  (M2) |
| --- | --- | --- | --- | --- | --- | --- | --- | --- | --- | --- | --- | --- | --- | --- | --- | --- | --- | --- | --- |
| 1 | 0.521 | 1 | 0.095 | 1 | 0.499 | 1 | 0.503 | 1 | 0.472 | 1 | 0.426 | 1 | 0.513 | 1 | 0.494 | 1 | 0.495 | 1 | 0.459 |
| 2 | 0.500 | 2 | 0.414 | 2 | 0.286 | 2 | 0.512 | 2 | 0.519 | 2 | 0.506 | 2 | 0.216 | 2 | 0.509 | 2 | 0.543 | 2 | 0.323 |
| 3 | 0.499 | 3 | 0.512 | 3 | 0.500 | 3 | 0.507 | 3 | 0.483 | 3 | 0.501 | 3 | 0.475 | 3 | 0.484 | 3 | 0.483 | 3 | 0.535 |
| 4 | 0.505 | 4 | 0.521 | 4 | 0.501 | 4 | 0.548 | 4 | 0.523 | 4 | 0.528 | 4 | 0.283 | 4 | 0.498 | 4 | 0.276 | 4 | 0.505 |
| 5 | 0.547 | 5 | 0.349 | 5 | 0.532 | 5 | 0.488 | 5 | 0.449 | 5 | 0.441 | 5 | 0.513 | 5 | 0.515 | 5 | 0.408 | 5 | 0.506 |
| 6 | 0.499 | 6 | 0.368 | 6 | 0.508 | 6 | 0.516 | 6 | 0.557 | 6 | 0.488 | 6 | 0.491 | 6 | 0.488 | 6 | 0.505 | 6 | 0.487 |
| 7 | 0.504 | 7 | 0.395 | 7 | 0.494 | 7 | 0.503 | 7 | 0.513 | 7 | 0.501 | 7 | 0.462 | 7 | 0.496 | 7 | 0.503 | 7 | 0.505 |
| 8 | 0.500 | 8 | 0.212 | 8 | 0.503 | 8 | 0.513 | 8 | 0.515 | 8 | 0.551 | 8 | 0.115 | 8 | 0.503 | 8 | 0.498 | 8 | 0.600 |
| 9 | 0.518 | 9 | 0.545 | 9 | 0.479 | 9 | 0.515 | 9 | 0.672 | 9 | 0.539 | 9 | 0.554 | 9 | 0.513 | 9 | 0.492 | 9 | 0.507 |
|  |  | 10 | 0.491 | 10 | 0.528 | 10 | 0.504 | 10 | 0.524 | 10 | 0.464 | 10 | 0.174 | 10 | 0.526 | 10 | 0.502 | 10 | 0.420 |
|  |  | 11 | 0.680 | 11 | 0.446 | 11 | 0.502 | 11 | 0.555 | 11 | 0.518 | 11 | 0.378 | 11 | 0.510 | 11 | 0.501 | 11 | 0.500 |
|  |  | 12 | 0.592 | 12 | 0.473 | 12 | 0.511 | 12 | 0.494 | 12 | 0.582 | 12 | 0.042 | 12 | 0.557 | 12 | 0.475 | 12 | 0.491 |
|  |  |  |  | 13 | 0.496 | 13 | 0.497 | 13 | 0.507 | 13 | 0.555 | 13 | 0.287 | 13 | 0.495 |  |  | 13 | 0.457 |
|  |  |  |  | 14 | 0.507 | 14 | 0.505 | 14 | 0.603 | 14 | 0.535 | 14 | 0.461 | 14 | 0.455 |  |  | 14 | 0.312 |
|  |  |  |  | 15 | 0.512 | 15 | 0.499 | 15 | 0.517 | 15 | 0.613 | 15 | 0.519 | 15 | 0.484 |  |  | 15 | 0.486 |
|  |  |  |  | 16 | 0.497 | 16 | 0.509 | 16 | 0.481 | 16 | 0.513 |  |  | 16 | 0.552 |  |  | 16 | 0.510 |
|  |  |  |  | 17 | 0.493 | 17 | 0.523 | 17 | 0.515 | 17 | 0.520 |  |  | 17 | 0.500 |  |  | 17 | 0.264 |
|  |  |  |  | 18 | 0.438 | 18 | 0.676 | 18 | 0.511 | 18 | 0.498 |  |  | 18 | 0.502 |  |  | 18 | 0.529 |
|  |  |  |  | 19 | 0.509 | 19 | 0.516 | 19 | 0.531 | 19 | 0.549 |  |  | 19 | 0.405 |  |  | 19 | 0.418 |
|  |  |  |  | 20 | 0.515 | 20 | 0.508 | 20 | 0.527 | 20 | 0.533 |  |  | 20 | 0.500 |  |  | 20 | 0.505 |
|  |  |  |  |  |  |  |  | 21 | 0.546 | 21 | 0.537 |  |  | 21 | 0.514 |  |  | 21 | 0.394 |
|  |  |  |  |  |  |  |  |  |  | 22 | 0.181 |  |  | 22 | 0.492 |  |  | 22 | 0.504 |
|  |  |  |  |  |  |  |  |  |  | 23 | 0.500 |  |  | 23 | 0.417 |  |  | 23 | 0.484 |
|  |  |  |  |  |  |  |  |  |  | 24 | 0.279 |  |  | 24 | 0.491 |  |  | 24 | 0.488 |
|  |  |  |  |  |  |  |  |  |  | 25 | 0.493 |  |  | 25 | 0.526 |  |  | 25 | 0.488 |
|  |  |  |  |  |  |  |  |  |  | 26 | 0.556 |  |  | 26 | 0.524 |  |  |  |  |
|  |  |  |  |  |  |  |  |  |  | 27 | 0.472 |  |  | 27 | 0.493 |  |  |  |  |
|  |  |  |  |  |  |  |  |  |  | 28 | 0.497 |  |  | 28 | 0.493 |  |  |  |  |
|  |  |  |  |  |  |  |  |  |  | 29 | 0.510 |  |  | 29 | 0.508 |  |  |  |  |
|  |  |  |  |  |  |  |  |  |  |  |  |  |  | 30 | 0.501 |  |  |  |  |
|  |  |  |  |  |  |  |  |  |  |  |  |  |  | 31 | 0.451 |  |  |  |  |
|  |  |  |  |  |  |  |  |  |  |  |  |  |  | 32 | 0.503 |  |  |  |  |
|  |  |  |  |  |  |  |  |  |  |  |  |  |  | 33 | 0.475 |  |  |  |  |
|  |  |  |  |  |  |  |  |  |  |  |  |  |  | 34 | 0.495 |  |  |  |  |
|  |  |  |  |  |  |  |  |  |  |  |  |  |  | 35 | 0.510 |  |  |  |  |
|  |  |  |  |  |  |  |  |  |  |  |  |  |  | 36 | 0.499 |  |  |  |  |

**S8. Observed (open circles) and predicted (solid line: posterior mean; dotted lines: 95% credible intervals) colony counts for all species and breeding colonies at which strong evidence for direct density dependence was detected**


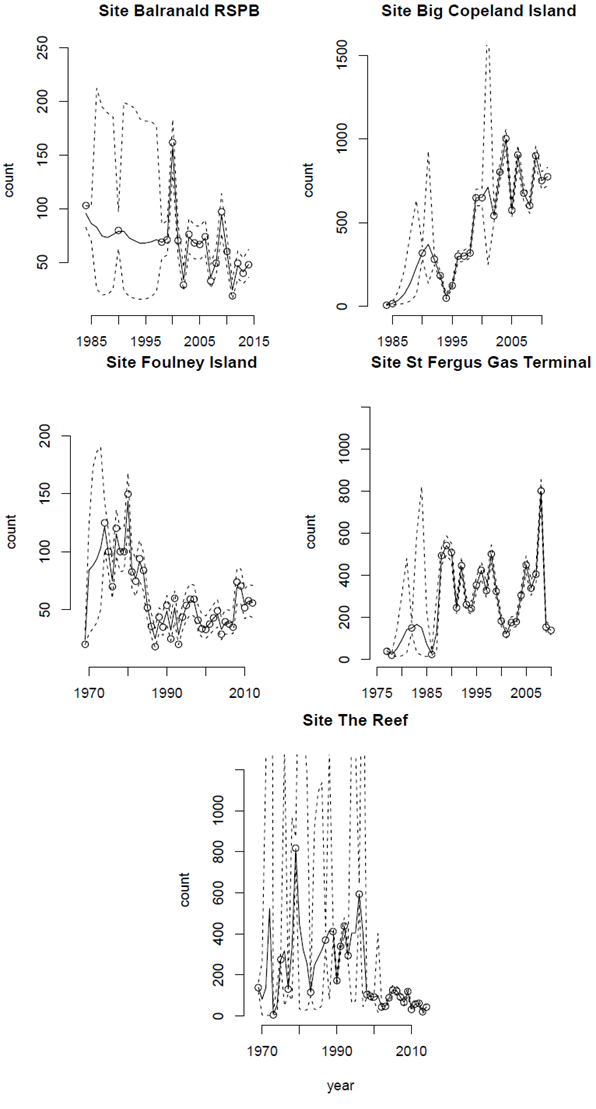


**Figure S8a.** Predicted (solid line) and 95% credible interval (dotted line) for population models fitted to Arctic tern colony counts (open circles) for the five colonies at which strong evidence for direct density dependence in population growth rate was detected (>95% posterior density was negative).


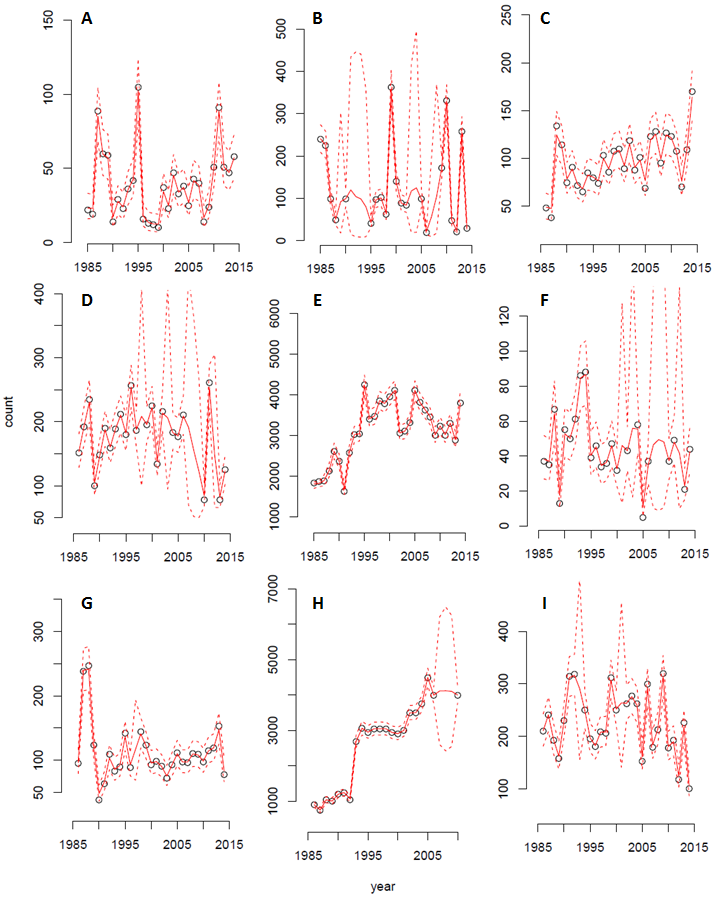
**Figure S8b.** Predicted (solid red line) and 95% credible interval (dotted red line) for population models fitted to Razorbill colony counts (open circles) for the nine colonies at which strong evidence for direct density dependence in population growth rate was detected (>95% posterior density was negative). Colony list: A: Caldey Island, B: Calf of Man, C: Fidra, D: great Orme, E: Isle of May, F: Little Orme, G: Saddle Point to Griffith Lorts Hole, H: Sanda Islands, I: St Bees Head.


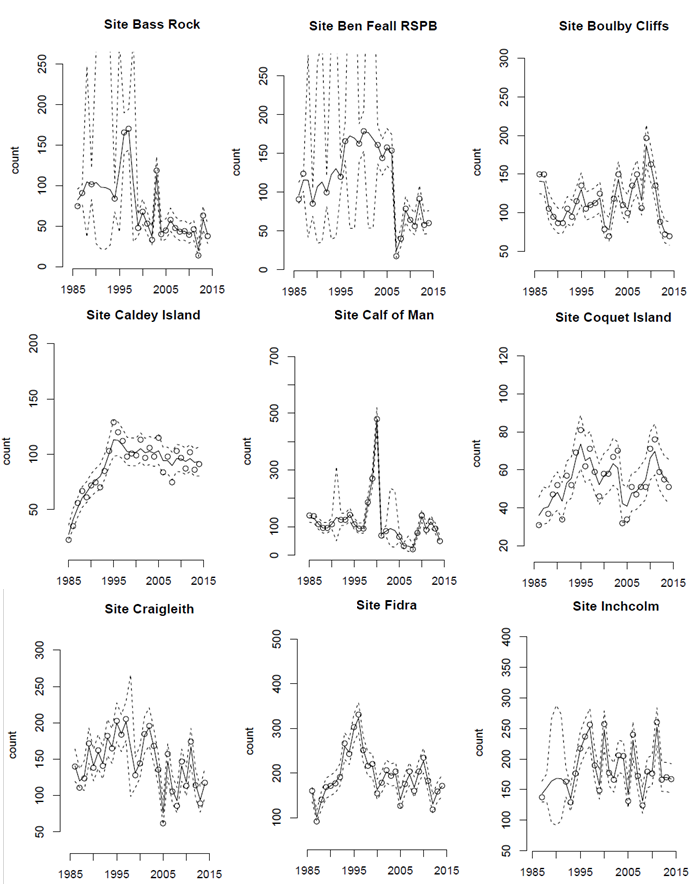


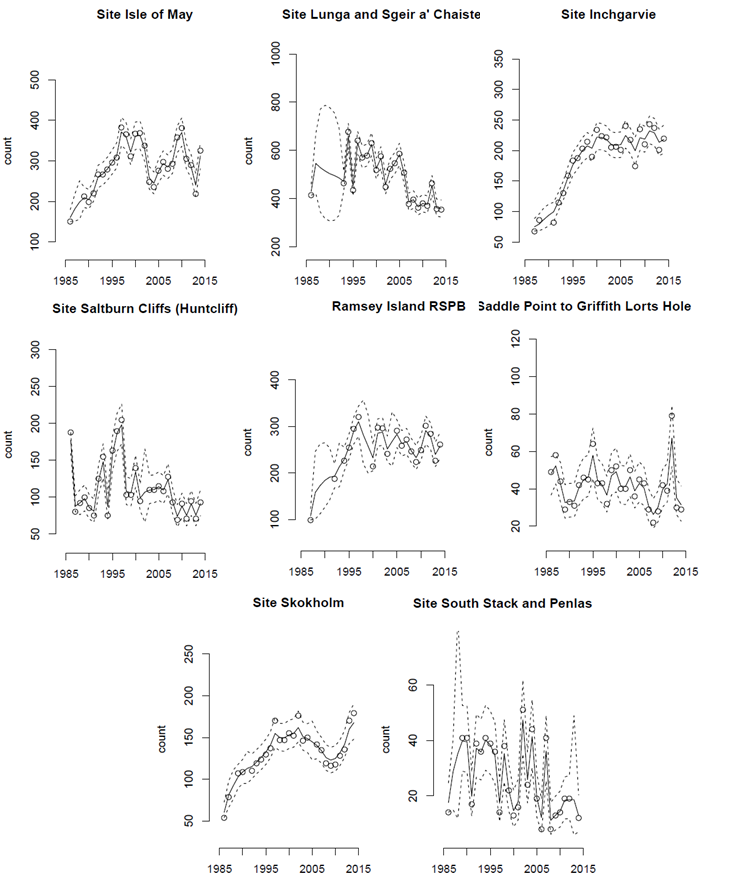


**Figure S8c.** Predicted (solid line) and 95% credible interval (dotted line) for population models fitted to Northern fulmar colony counts (open circles) for the 17 colonies at which strong evidence for direct density dependence in population growth rate was detected (>95% posterior density was negative).


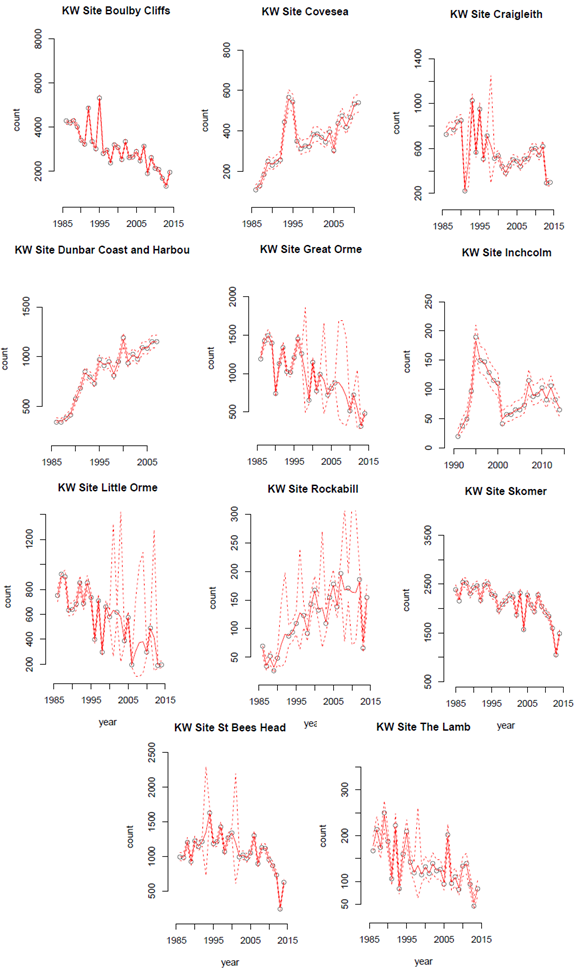
**Figure S8d.** Predicted (solid red line) and 95% credible interval (dotted red line) for population models fitted to Black-legged kittiwake colony counts (open circles) for the eleven colonies at which strong evidence for direct density dependence in population growth rate was detected (>95% posterior density was negative).


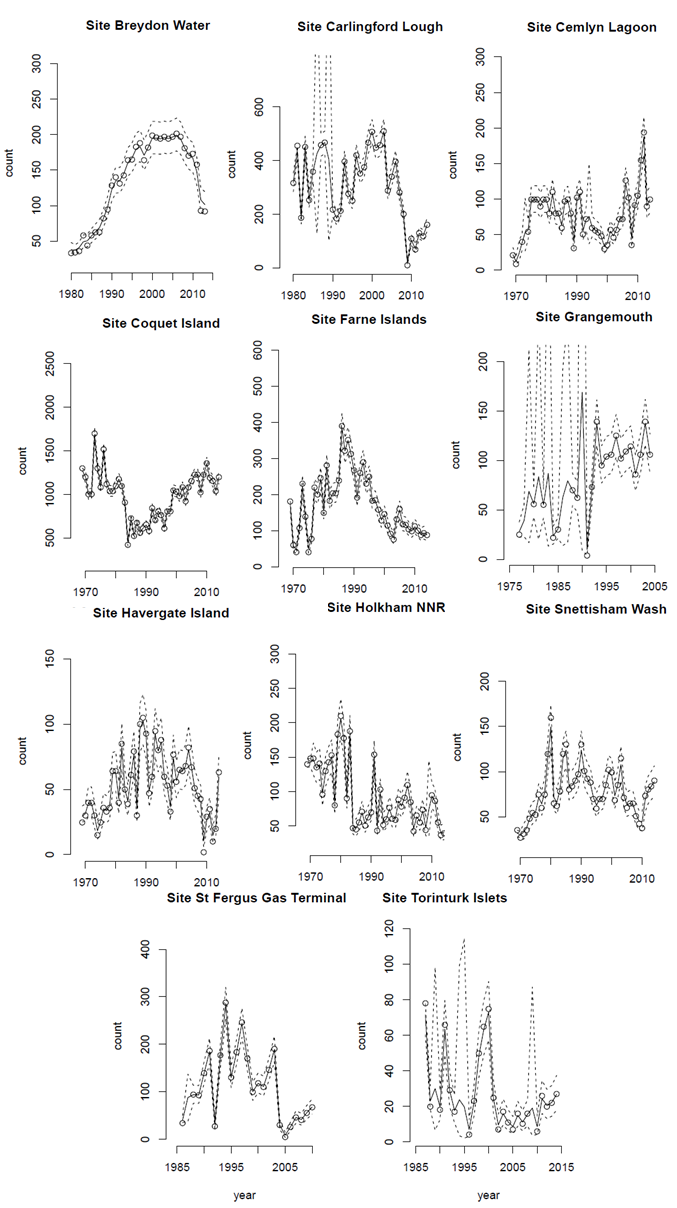


**Figure S8e.** Predicted (solid line) and 95% credible interval (dotted line) for population models fitted to Common tern colony counts (open circles) for the 11 colonies at which strong evidence for direct density dependence in population growth rate was detected (>95% posterior density was negative).


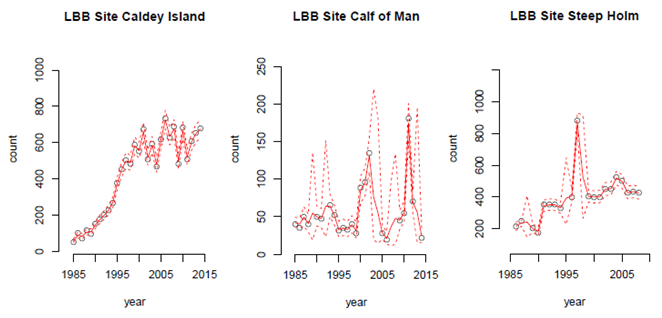


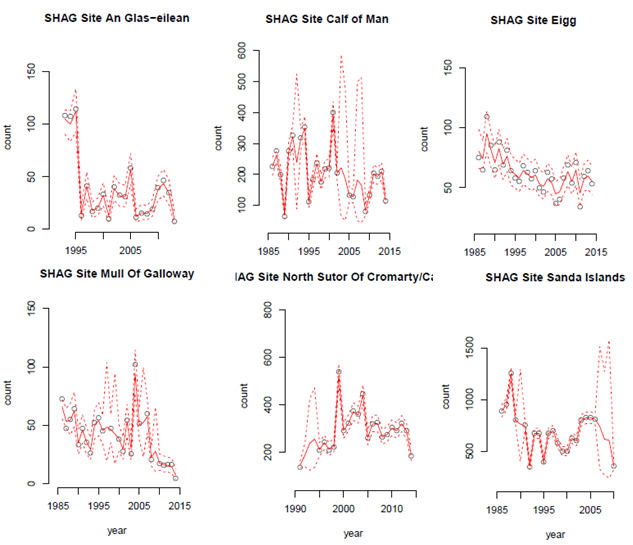
**Figure S8f.** Predicted (solid red line) and 95% credible interval (dotted red line) for population models fitted to Lesser black-backed gull colony counts (open circles) for the three colonies at which strong evidence for direct density dependence in population growth rate was detected (>95% posterior density was negative).

**Figure S8g.** Predicted (solid red line) and 95% credible interval (dotted red line) for population models fitted to European shag colony counts (open circles) for the six colonies at which strong evidence for direct density dependence in population growth rate was detected (>95% posterior density was negative).


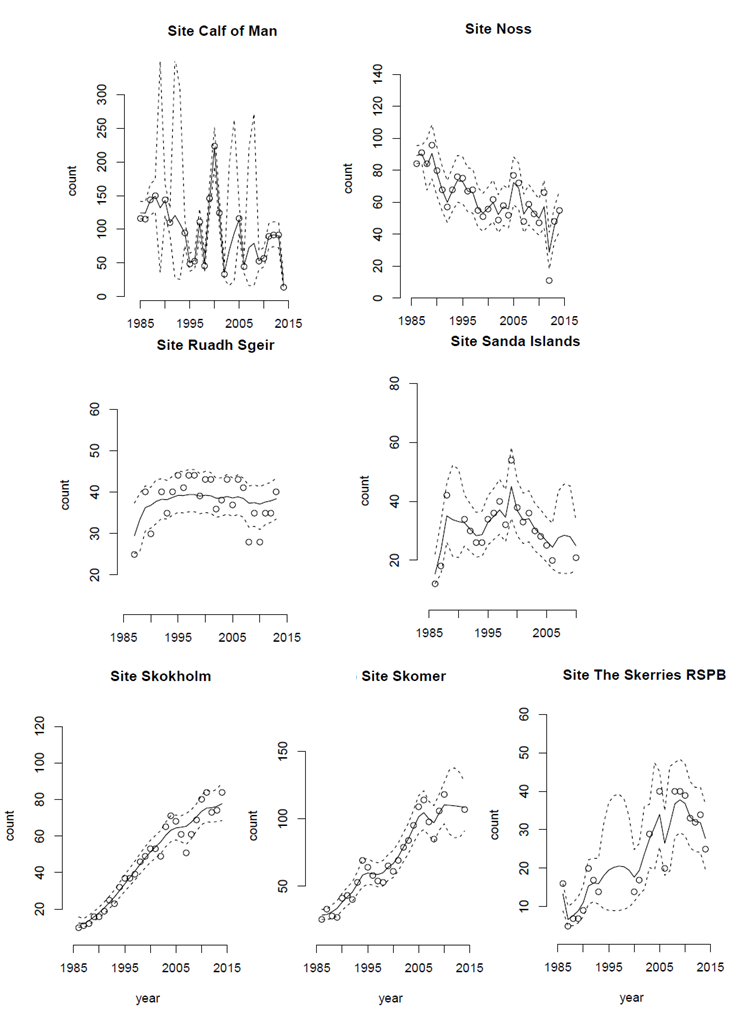


**Figure S8h.** Predicted (solid line) and 95% credible interval (dotted line) for population models fitted to Great black-backed gull colony counts (open circles) for the 7 colonies at which strong evidence for direct density dependence in population growth rate was detected (>95% posterior density was negative).
